# Role of Nesprin-2 and RanBP2 in BICD2-associated brain developmental disorders

**DOI:** 10.1371/journal.pgen.1010642

**Published:** 2023-03-17

**Authors:** Julie Yi, Xiaoxin Zhao, Crystal R. Noell, Paige Helmer, Sozanne R. Solmaz, Richard B. Vallee

**Affiliations:** 1 Department of Pathology and Cell Biology, Columbia University Medical Center, New York, New York, United States of America; 2 Department of Chemistry, Binghamton University, Binghamton, New York, New York, United States of America; Fred Hutchinson Cancer Research Center, UNITED STATES

## Abstract

Bicaudal D2 (BICD2) is responsible for recruiting cytoplasmic dynein to diverse forms of subcellular cargo for their intracellular transport. Mutations in the human *BICD2* gene have been found to cause an autosomal dominant form of spinal muscular atrophy (SMA-LED2), and brain developmental defects. Whether and how the latter mutations are related to roles we and others have identified for BICD2 in brain development remains little understood. BICD2 interacts with the nucleoporin RanBP2 to recruit dynein to the nuclear envelope (NE) of Radial Glial Progenitor cells (RGPs) to mediate their well-known but mysterious cell-cycle-regulated interkinetic nuclear migration (INM) behavior, and their subsequent differentiation to form cortical neurons. We more recently found that BICD2 also mediates NE dynein recruitment in migrating post-mitotic neurons, though *via* a different interactor, Nesprin-2. Here, we report that Nesprin-2 and RanBP2 compete for BICD2-binding *in vitro*. To test the physiological implications of this behavior, we examined the effects of known *BICD2* mutations using *in vitro* biochemical and *in vivo* electroporation-mediated brain developmental assays. We find a clear relationship between the ability of BICD2 to bind RanBP2 *vs*. Nesprin-2 in controlling of nuclear migration and neuronal migration behavior. We propose that mutually exclusive RanBP2-BICD2 *vs*. Nesprin-2-BICD2 interactions at the NE play successive, critical roles in INM behavior in RGPs and in post-mitotic neuronal migration and errors in these processes contribute to specific human brain malformations.

## Author summary

Mutations in *BICD2* gene have been found to cause brain developmental defects, as well as other disorders, like Spinal Muscular Atrophy with Lower Extremity Dominance 2 (SMA-LED2). Despite its impact on human disorders, the underlying cause of *BICD2* associated disease conditions remains incompletely understood. During brain development, BICD2 recruits dynein to the nuclear envelope of pre- and post-mitotic neuronal cells *via* successive interactions with two different nuclear envelope proteins, RanBP2 and Nesprin-2. How the switch in BICD2-dynein cargo interactions is achieved and its physiological necessity have emerged as important questions for understanding normal and abnormal brain development. To answer the questions, we first performed biochemical assays to compare RanBP2 and Nesprin-2 bindings to BICD2. We find that RanBP2 and Nesprin-2 bind to distinct sites within BICD2 region, but their interactions are mutually exclusive. Second, we test the relative physiological roles of the two mechanisms by expressing disease-causing BICD2 variants in embryonic rat brains. We find that BICD2’s ability to bind RanBP2 *vs*. Nesprin-2 controls specific brain developmental steps, and defects in the binding to these NE proteins contribute to the specific brain deformities associated with *BICD2* mutations.

## Introduction

Bicaudal D (BICD) is a well-known developmental gene found to link cytoplasmic dynein to diverse forms of subcellular cargo in *Drosophila melanogaster* [1–2] and vertebrates [3–7]. BICD also serves as a scaffold for co-assembly of dynein with its regulators dynactin and LIS1 [8, 9], to form a mechanochemical supercomplex [10].

**Fig 1:**
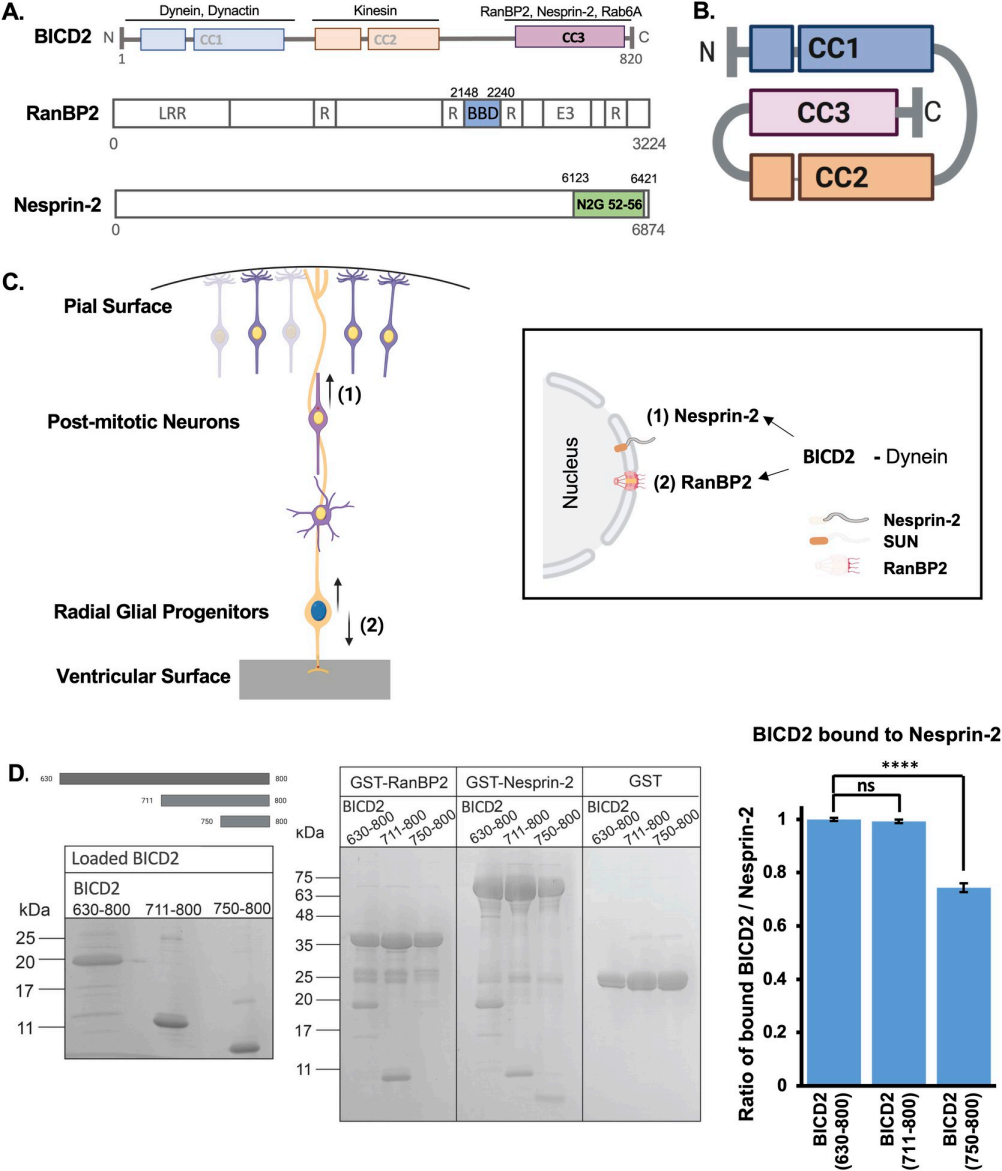
Structural organization and interactions of BICD2, RanBP2, and Nesprin-2. (A) Schematic representation of BicD2 (top), RanBP2 (middle), and Nesprin-2 (bottom). BicD2 (top): The colored boxes represent the three coiled-coil domains (CC1, CC2, and CC3) of the BICD2 protein. Interactors for each domain are noted on top. RanBP2 (Middle): A drawing of RanBP2 (modified from [14]), showing leucine-rich region (LRR), an E3 Sumo ligase domain (E3), and four Ran-binding domains (R), two of which flanking the BicD2 binding domain (BBD). “BBD” (highlighted in blue) of RanBP2 is used in the biochemical assays of this study. Nesprin-2 (bottom): A drawing of Nesprin-2 [3, 21] with “N2G 52–56” highlighted in green. The BicD2 binding “N2G 52–56” fragment [3] is used in the biochemical assays of this study. (B) Schematic depiction of BICD2 in an autoinhibited state. (C) Drawing of the cerebral cortex during development (Left). Both the apical nuclear migration of Radial Glial Progenitors and the basal migration of post-mitotic neurons are mediated by dynein, which is recruited to the nuclear envelope via two distinct pathways: (2) RanBP2- or (1) Nesprin-2-BICD2 interactions (Right). (D) Left panel: BICD2 fragments used in the GST pull-down (Top left). A Coomassie-stained SDS-PAGE of the purified BICD2 fragments used in the GST pull-down assay is shown below. Middle panel: An SDS-PAGE of the elution fractions of the GST-pulldown assay is shown. The GST-tagged RanBP2 fragment (BICD2 binding domain, aa 2148–2240) pulls down the BICD2 fragments aa 630–800 and aa 711–800, but not the fragment aa 750–800. The GST-tagged Nesprin-2 fragment (BICD2/Dynein binding domain, for the sequence see S1A Fig) pulls down each of the three BICD2 fragments. A pull-down assay with GST is shown as a negative control. The molar masses of standards are indicated on the left of each SDS-PAGE. Right panel: The intensities of the BICD2 gel bands from the pull-down assays with Nesprin-2 were quantified from 3 sets of experiments, a representative dataset is shown in S1B Fig. Discrete amounts of the three BICD2 fragments were analyzed on the SDS-PAGE and used as calibration standards to calculate the amounts of BICD2 bound to Nesprin-2 (μg) from the pull-down assays. Since the staining intensities are directly proportional to the molar masses of each fragment, in order to compare the number of bound BICD2 molecules, the BICD2 amounts were converted to the molar concentration divided by the molar concentration of Nesprin-2 and normalized to 1 for the longest fragment. The error was calculated as the standard deviation. Student’s t-test was performed against the BICD2 (aa 630–800) condition and the statistical significance is shown. (**** p<0.0001 and ns = not significant).

*D*. *melanogaster* BICD was initially identified as a polarity factor, which was later found to play an essential role in mRNA localization, facilitated by BICD*-*mediated interactions with Egalitarian [11] and Fragile X Mental retardation protein (FMRP) [12]. The mammalian homolog BICD2 was subsequently found to interact with a number of “cargo” proteins, including Rab6A [6], which links dynein [4] and kinesin to exocytic vesicles in cultured mammalian cells [13]. BICD2 (Fig 1A, top) was more recently found to recruit dynein to the nucleoporin RanBP2 (Fig 1A, middle) [7] and, by our lab, to the LINC complex protein Nesprin-2 at the nuclear envelope (NE) surface (Fig 1A, bottom) [3], consistent with important roles for BICD2 in vertebrate brain development. We found that RanBP2 and Nesprin-2 play key roles in mediating nuclear migration in proliferating neuronal precursors (RGPs) [5, 14] and migrating neurons [3], respectively.

BICD2 is one of four vertebrate BICD orthologues, which also include *BICD1*, *BICDR-1*, and *BICDR-2*. The *BICD2* gene encodes a 95 kDa polypeptide with a substantial coiled-coil α-helical structure (Fig 1A, top). The coiled-coil 1 (CC1) and coiled-coil 2 regions (CC2) interact, respectively, with cytoplasmic dynein/dynactin [15] and kinesin [13]. The coiled-coil 3 region of BICD2 (CC3) serves in cargo binding [4–7, 9]. When not bound to cargo, BICD2 adopts an autoinhibited conformation involving an intramolecular interaction between CC1 and CC3 (Fig 1B) that masks the dynein/dynactin binding site, thus preventing dynein recruitment [4, 9, 16–18]. Autoinhibition, in turn, has been proposed to be regulated by another unusual BICD2 feature, a coiled-coil registry-shift within CC3, which involves a vertical displacement of the two α-helices against each other by ~one helical turn [16, 19] to form another distinct coil-coil structure (see further in discussion). Additionally, BICD2 and dynein interaction was also found to be under the control of the Cyclin-dependent kinase 1 (CDK1) and the Polo-family kinase 1 (PLK1) phosphorylation of BICD2 [20].

To date, 22 independent disease-causing mutations have been reported at sites throughout the human *BICD2* coding region [22–36], revealing a range of clinical phenotypes. Most of *BICD2* mutations have been found to cause Spinal Muscular Atrophy with Lower Extremity Dominance 2 (SMA-LED2), characterized by slowly progressive muscle wasting, and weakness in the lower extremities [25–27, 33]. An impaired Rab6A mediated secretion has been shown in the SMA-LED2 patient fibroblasts [37]. But, the underlying molecular mechanisms leading to the disease phenotype still remain elusive, particularly owing to an incomplete understanding of BICD2’s roles in the neuromuscular system.

Our lab had identified multiple roles for cytoplasmic dynein, BICD2 and other dynein regulators and accessory factors in rat brain development. We found clear, important roles for BICD2 in neurogenesis in the radial glial progenitor cells, as well as in subsequent neuronal migration during specific developmental stages [3,5,14]. Consistent with these findings, so far, three different *BICD2* mutations have been reported to cause developmental brain diseases [22,28,35].

During brain development, the nucleus in Radial Glial Progenitor (RGP) has long been known to oscillate away from and back to the ventricular surface of the embryonic brain as these cells multiply and begin to differentiate to form postmitotic neurons (Fig 1C; Left). The latter then travel to the cortical plate (CP), where they become mature neurons. We found that BICD2 plays a central role in dynein recruitment to the nuclear envelope (NE) in both RGPs and postmitotic neurons. In particular, knockdown of BICD2 in embryonic rat brain impaired both dynein-mediated apical nuclear migration in the RGP cells, as well as migration of post-mitotic neurons to the CP [5]. We found BICD2 itself to be recruited to the NE in these cells *via* two distinct mechanisms: BICD2 binds to the RGP NE *via* the nucleoporin RanBP2 [5,14], but to the NE of post-mitotic neurons *via* the LINC complex component Nesprin-2 [3] (Fig 1C; Right).

The physiological roles of BICD2 In the developing brain have also been assessed in a knock-out mouse model, which displayed hydrocephalus and cerebellar malformation, the latter was proposed to result from impaired secretion of Tenascin-C by the cerebellar Bergman Glial cells [38]. Also, cortical expression of the polymicrogyria-causing BICD2 R694C mutation caused delayed migration of postmitotic neurons within the cortical plate [39], but the underlying molecular defect causing the delay remains unknown. More recently, a C-terminally truncated Lissencephaly-associated form of BICD2 (K775X) was found to disrupt Nesprin-2 binding and to cause a severe defect in post-mitotic neuronal migration in mouse cerebral cortex [35]. But its effect on RanBP2 mediated function or interaction has not been studied.

As yet, only a small number of known *BICD2* mutations have been implicated in brain developmental disorders, and there is yet relatively little evidence linking genotype and phenotype. In this study, we address this issue by focusing on the effect of BICD2 mutations on the interaction between BICD2 and its two NE associated interactors. We provide the first evidence identifying the Nesprin-2 binding site within BICD2, which proves to be distinct from, though partially overlapping with that for RanBP2. We also show a clear correlation between BICD2 mutations that preferentially bind Nesprin-2- vs. RanBP2- with specific developmental defects in the embryonic rat brain, providing important new guidance linking phenotype with genotype. We also sought to identify BICD2 mutations that might selectively interfere with the BICD2-RanBP2 vs. BICD2-Nesprin-2 interactions in the developing brain, to help distinguish the specific developmental contributions of altered INM *vs*. post-mitotic neuronal migration in the developing brain. Our findings have potentially important implications for understanding BICD2 pathophysiology and the mechanisms responsible for normal cortical development.

## Results

### Overlapping but distinct Nesprin-2 *vs*. RanBP2 binding to regions within the BICD2 CC3 domain

Whereas the BICD2 binding site for RanBP2 is known [18], we now sought to determine that for Nesprin-2. For this analysis, we expressed the portion of a Nesprin-2 shown to bind both dynein and BICD2, termed “N2G SR 52–56” [3, 21] (Fig 1A, bottom) referred to simply as “Nesprin-2” hereafter (see S1A Fig for the protein sequence). This construct was used for binding assays with three bacterially expressed BICD2 fragments: BICD2 aa 630–800, which contains the entire predicted CC3 domain; BICD2 (aa 711–800); and BICD2 (aa 750–800) (Fig 1D). The purified BICD2 fragments were incubated *in vitro* with GST-tagged Nesprin-2 and tested by pull-down assay for a direct interaction. All three of the BICD2 fragments interacted with Nesprin-2. A quantification of the molar concentration of these three BICD2 fragments bound to Nesprin-2 (Fig 1D and S1B Fig) showed that the amount of bound BICD2 aa 750–800 molecules is approximately 73% compared to the longer fragments (aa 630–800 and 711–800). These results suggest that the critical binding residues for Nesprin-2 reside within the aa 750–800 region (Fig 1D). Unlike Nesprin-2, however, the BICD2-binding RanBP2 fragment (aa 2148–2240) [7, 14] (Fig 1A, middle), referred to as “RanBP2” hereafter, showed binding to the longer BICD2 fragments aa 630–800 and aa 711–800, but not the shorter aa 750–800 fragment (Fig 1D), consistent with a previous study [18]. We note that the binding of RanBP2 to a BICD2 fragment lacking the aa 750–800 could not be tested because the deletion of aa 750–800 caused destabilization of the coiled-coil structure in BICD2 CC3. Together, these results suggested that the BICD2 interaction residues for RanBP2 and Nesprin-2 must to some extent differ.

**Fig 2:**
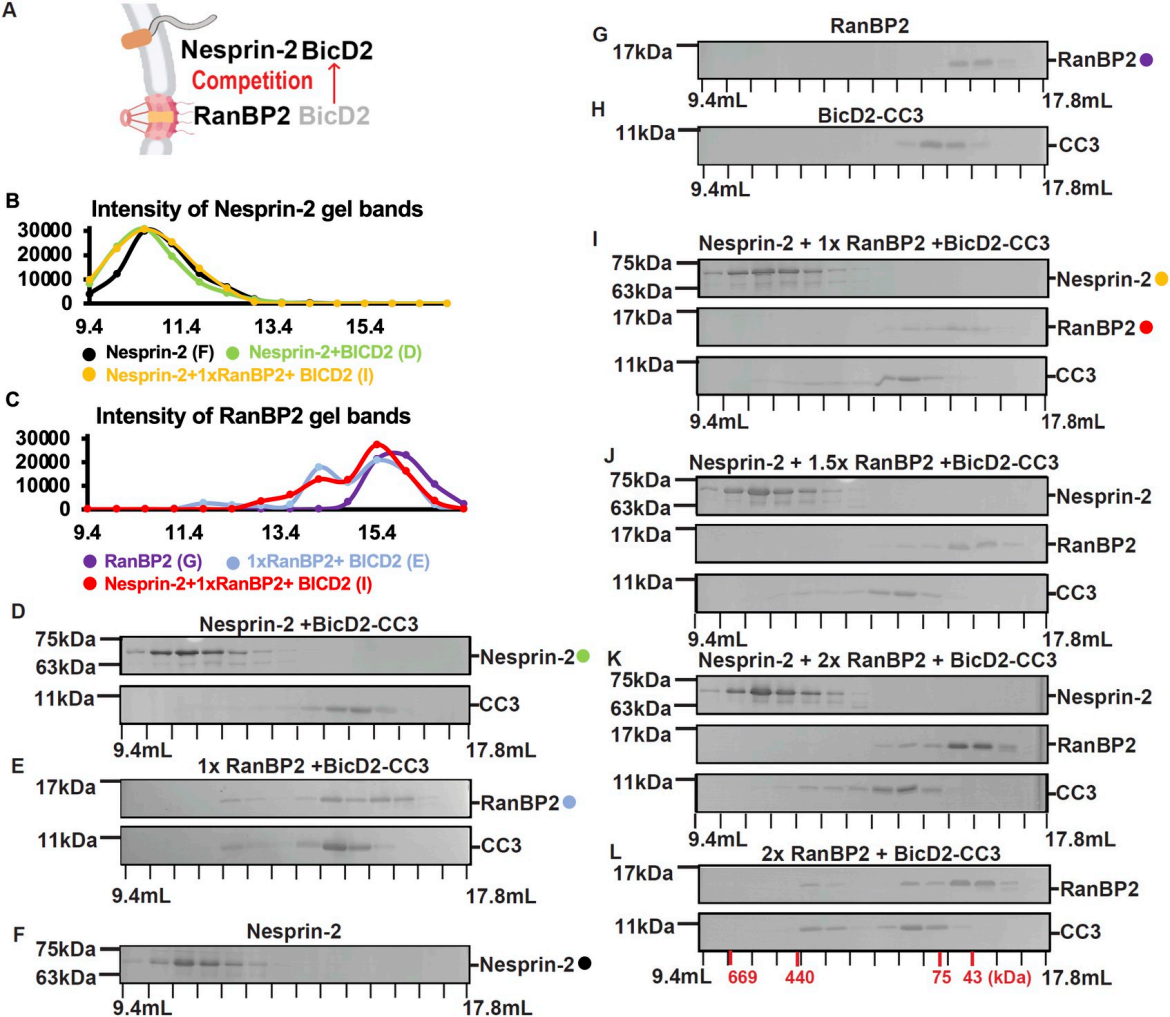
Nesprin-2 competes with RanBP2 for BICD2-CC3 binding. (A) Schematic representation of the assay. (B-L) A purified RanBP2 fragment (aa 2148–2240) and/or Nesprin-2 fragment (for sequence see S1A Fig) plus BICD2-CC3 (residues 711–800) were mixed at a 1:1:1 molar ratio and analyzed for physical interactions by size exclusion chromatography. SDS-PAGE analysis of the elution fractions (D-L) and (B,C) intensity profiles of the gel bands are shown. (B) Intensity profiles of Nesprin-2 gel bands from the SDS-PAGEs shown in panels D, F and I (see color-coded circles). (C) Intensity profiles of RanBP2 gel bands from the SDS-PAGEs shown in panels E, G and I (see color-coded circles). (D-L) SDS-PAGE of elution fractions for: (D) Nesprin-2 and BICD2-CC3 (E) RanBP2 and BICD2-CC3. (F, G, H) As controls, the individual proteins were also analyzed: (F) Nesprin-2; (G) RanBP2; (H) BICD2-CC3. (I, J, K) Nesprin-2, RanBP2 and BICD2-CC3 were mixed in 1:1:1 molar ratio, in 1:1.5:1 molar ratio and 1:2:1 molar ratio with increasing amounts of RanBP2. (L) As a control for (K), RanBP2 and BICD2-CC3 were mixed at a 2:1 molar ratio. For all gels, the masses of molecular weight standards are indicated at left and elution volumes at the bottom. All experiments were repeated at least twice. The elution volumes of molar mass standards are indicated in red at the bottom of panel (L).

In view of these data, we tested whether BICD2 can accommodate both NE-binding cargo proteins at once (Fig 2A). We note that the RanBP2 fragment (aa 2148–2240) is the minimal BICD2-binding fragment, which can bind to the full-length BICD2 to form processive dynein/dynactin/BICD2/RanBP2 complexes in *in vitro* assays [40]. Pairwise mixtures of purified RanBP2 fragment (Fig 1A, middle) and Nesprin-2 fragment (Fig 1A, bottom) plus the aa 711–800 BICD2 fragment (now termed “BICD2-CC3”) were allowed to interact in solution, then analyzed for complex formation by size-exclusion chromatography (Fig 2). The BICD2-CC3 fragment showed clear evidence of binding to Nesprin-2 (Fig 2B and Fig 2D), as indicated by its early elution compared to the individual BICD2-CC3 protein (Fig 2B and compare Fig 2D with Fig 2F and 2H).

The experiment was repeated with the RanBP2 fragment, which also clearly interacted with BICD2-CC3, since the elution volumes of both RanBP2 and BICD2-CC3 were shifted towards higher mass when the complex was analyzed compared to the individual proteins (Fig 2C and compare Fig 2E with Fig 2G, and 2H). We have previously determined the molar masses of the RanBP2/BICD2-CC3 complex and the individual proteins by size-exclusion chromatography coupled to multi-angle light scattering, which is highly accurate (<5% error) and by small-angle X-ray scattering. BICD2-CC3 predominantly forms a dimer with a molar mass of 21.8 kDa, RanBP2 forms a monomer with a mass of 10.6 kDa, and the RanBP2/BICD2-CC3 forms a 2:2 complex with a mass of 43.0 kDa [41, 42]. It should be noted that RanBP2 is an intrinsically disordered protein, while BICD2-CC3 and the RanBP2/BICD2-CC3 complex form elongated conformations, and therefore all three samples elute earlier from a size exclusion chromatography column compared to globular proteins with the same molar mass, such as the molar mass standards that were used for calibration of the column. Notably, in the mixture with all three protein fragments, Nesprin-2 exhibited a strong interaction with BICD2-CC3 (Fig 2I). The elution peak of BICD2-CC3 was shifted towards higher mass and co-eluted with Nesprin-2, while the RanBP2 elution peak was comparable to that of the RanBP2 alone control and not shifted to higher mass (Fig 2G). This result suggests that Nesprin-2 outcompetes RanBP2 for BICD2 binding. To examine further the competition between RanBP2 and Nesprin-2 for BICD2 binding, RanBP2 was added to the Nesprin-2-BICD2-CC3 mixture at a range of concentrations corresponding to a 1, 1.5- or 2-fold molar excess. Again, BICD2-CC3 predominantly interacted with Nesprin-2, and only a small fraction of BICD2-CC3 retained its interaction with RanBP2 (Fig 2J–2L), suggesting that Nesprin-2 has a higher affinity for BICD2 than does RanBP2. Taken together, our data suggest that 1) RanBP2 and Nesprin-2 interact with BICD2, through overlapping but distinct sites within the BICD2 CC3 domain. 2) Despite differences in BICD2 binding, RanBP2 and Nesprin-2 still compete for BICD2 binding, though Nesprin-2 outcompetes RanBP2.

### The human disease-causing *BICD2* mutations R694C and E774G exhibit abnormally enhanced RanBP2 *vs*. Nesprin-2 binding, respectively

As noted earlier, pathogenic BICD2 mutations have been identified throughout the molecule, most reported to cause SMA-LED2. Intriguingly, three mutations, so far, have also been found to cause brain developmental abnormalities as well, including cerebellar hypoplasia and the cortical malformations polymicrogyria and lissencephaly [22, 28, 35], expanding the range of BICD2 mutant phenotypes beyond those associated with the neuromuscular system.

**Fig 3:**
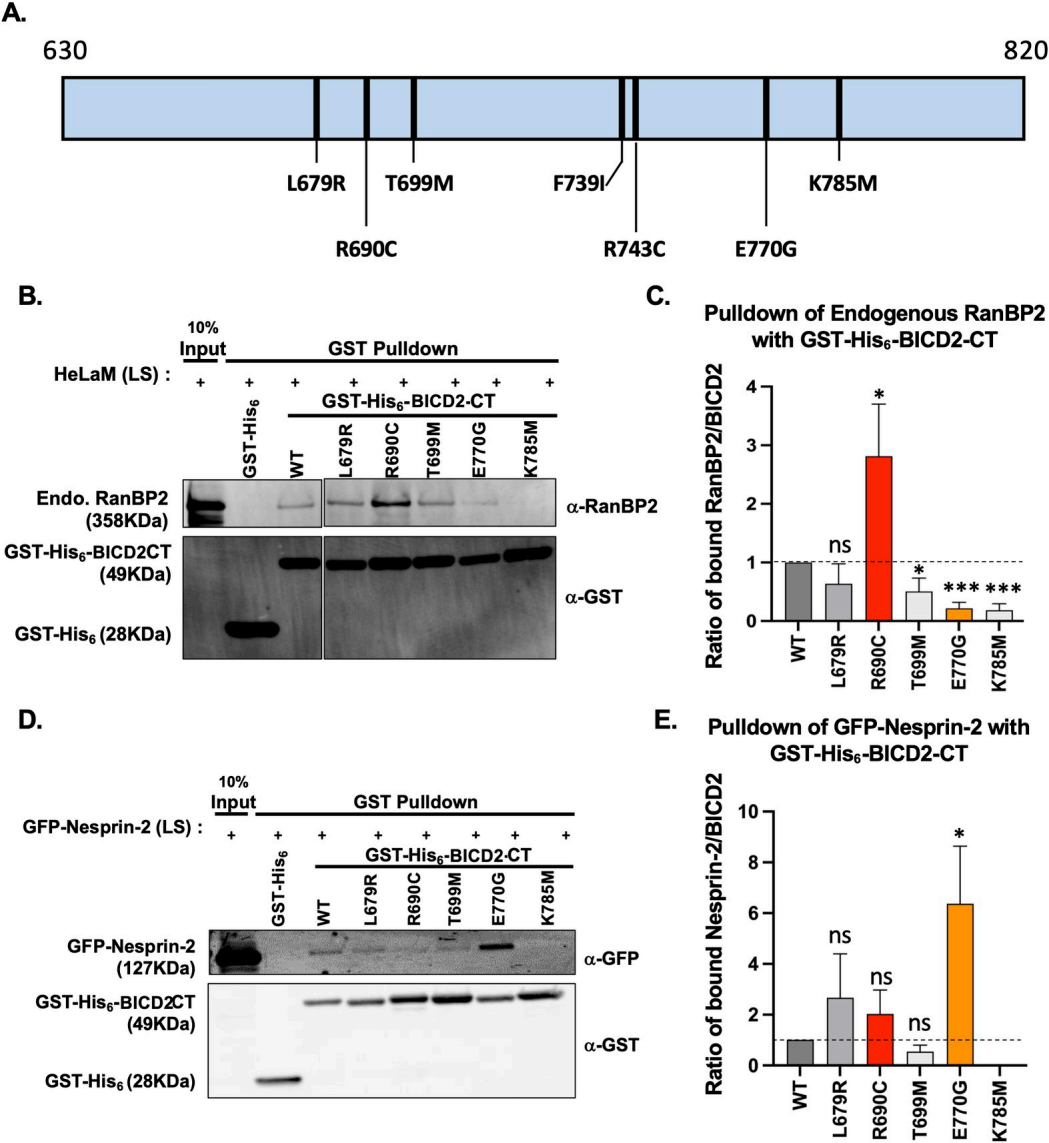
Human BICD2 mutations with defects in RanBP2 and/or Nesprin-2 interactions. (A) Depiction of BICD2-CT (aa 630–820, note BICD2 CC3 fragment is aa 711–800) with each missense mutation marked (*black bars)*. Human disease causing mutations of BICD2 (L679R, R690C, T699M, and E770G in mouse *Bicd2*), one *Drosophila* mutation (K785M in mouse *Bicd2*) and the registry-shift locking mutations (F739I and R743C) are marked to show their relative positions within the BICD2 CT region. (B-E) GST-His_6_-BICD2 CT WT and missense mutant forms of this construct were used in pull-downs from HeLaM cell lysates. The co-precipitated proteins were identified by immunoblotting against anti-RanBP2 or anti-GFP to detect GFP-Nesprin-2 and GST for the BICD2 CT proteins. (B) Immunoblot of GST pulldown from untransfected HeLaM cell lysate using anti-RanBP2 and anti-GST antibodies. (C) Ratio of the RanBP2 signal intensity to the GST signal is normalized to the wildtype control. (D) Western blot of GST pulldown from GFP-Nesprin-2 expressing HeLaM cell lysate, immunoblotted with anti-GFP and anti-GST antibodies. (E) Ratio of the GFP signal intensity to the GST signal is normalized to the wildtype control. We note that GFP-Nesprin-2 was not detectable in the pulldown for K785M mutation. n = 4 for (B,C) and n = 3 for (D,E); All error bars are S.E.M. Student’s t-test was performed against the wildtype control. (* p<0.05; ** p <0.01; *** p<0.001; ns = not significant).

To test whether and how the human *BICD2* mutations, in particular those associated with altered brain development, might affect the BICD2-RanBP2 or BICD2–Nesprin-2 interaction, we screened four missense variants of human *BICD2* (residue numbering of mouse *Bicd2*) L683R (L679R), R694C (R690C), T703M (T699M), and E774G (E770G) (S2 Fig) and a well-known lethal mutation of *D*. *melanogaster* BICD K730M (K785M in mouse *Bicd2*) within the C-terminal domain of mouse *Bicd2* (Fig 3A). The L679R and R690C mutations have been reported to cause brain abnormalities [22, 28], whereas T699M [25] and E770G [27] cause SMA-LED2 without any reported defects in the brain. A human BICD2 K775X mutation [35] was reported only after our brain developmental studies had been carried out, and it was not included in the current study. For the other mutations, we expressed and purified recombinant mutant and wildtype forms of GST-His_6_-BICD2-CT (aa 630–820) for use in pull-down assays from untransfected or a GFP tagged Nesprin-2 fragment (“GFP-Nesprin-2”)-expressing HeLaM cell lysates (Fig 3B–3E). Given a reported delay in neuronal redistribution to the cortical plate in BICD2 R690-expressing mouse brain [39], which should involve Nesprin-2-BICD2 mediated NE-dynein recruitment [3], we initially hypothesized that the R690C mutation might act to reduce the affinity of BICD2 for GFP-Nesprin-2. To our surprise, our BICD2-CT R690C mutant fragment showed no significant change in affinity for GFP-Nesprin-2, but instead, an ~2.8-fold increase in RanBP2 binding (Fig 3B and 3C). Because RanBP2 and Nesprin-2 compete for BICD2 binding (Fig 2), we reasoned that the enhanced RanBP2 binding might interfere with the BICD2-Nesprin-2 interaction in *in vivo* setting, explaining the defect in Nesprin-2-BICD2-mediated function observed in an earlier brain study [39]. Moreover, BICD2-CT E770G showed a markedly reduced degree of endogenous RanBP2 binding (Fig 3B and 3C), though a ~6-fold increase in GFP-Nesprin-2 binding (Fig 3D and 3E). The Cerebellar hypoplasia-causing BICD2-CT L679R mutation showed no detectable change in binding to RanBP2 or Nesprin-2, which is consistent with the relatively mild clinical phenotype associated with this mutation [22]. The mouse equivalent of the lethal *Drosophila-*mutation, K785M, resulted in almost no binding to either RanBP2 or Nesprin-2 (Fig 3B–3E). Last, the SMA-LED2-causing T699M mutation resulted in a mild reduction in binding to RanBP2 and no change in Nesprin-2 binding. Together, these data demonstrate *BICD2* mutational site-specific effects on RanBP2- or Nesprin-2- binding. In particular, the R690C and the E770G showed surprisingly enhanced binding to RanBP2 or Nesprin-2, respectively, representing a *gain-of-function*.

### INM *vs*. *neuronal migration-specific effects of BICD2* E770G and *BICD2* R690C mutations

From the biochemical screening of *BICD2* mutations described above, we identified two apparent *gain-of-function* mutations, R690C and E770G, which result in greatly enhanced binding of BICD2 to RanBP2 *vs* Nesprin-2, respectively. Moreover, BICD2 E770G, which increased Nesprin-2 binding (Fig 3D and 3E), showed markedly reduced binding to RanBP2 (Fig 3B and 3C), consistent with the mutually exclusive interaction characteristics we observed for Nesprin-2 and RanBP2 with the BICD2 CC3 domain, as described above.

**Fig 4:**
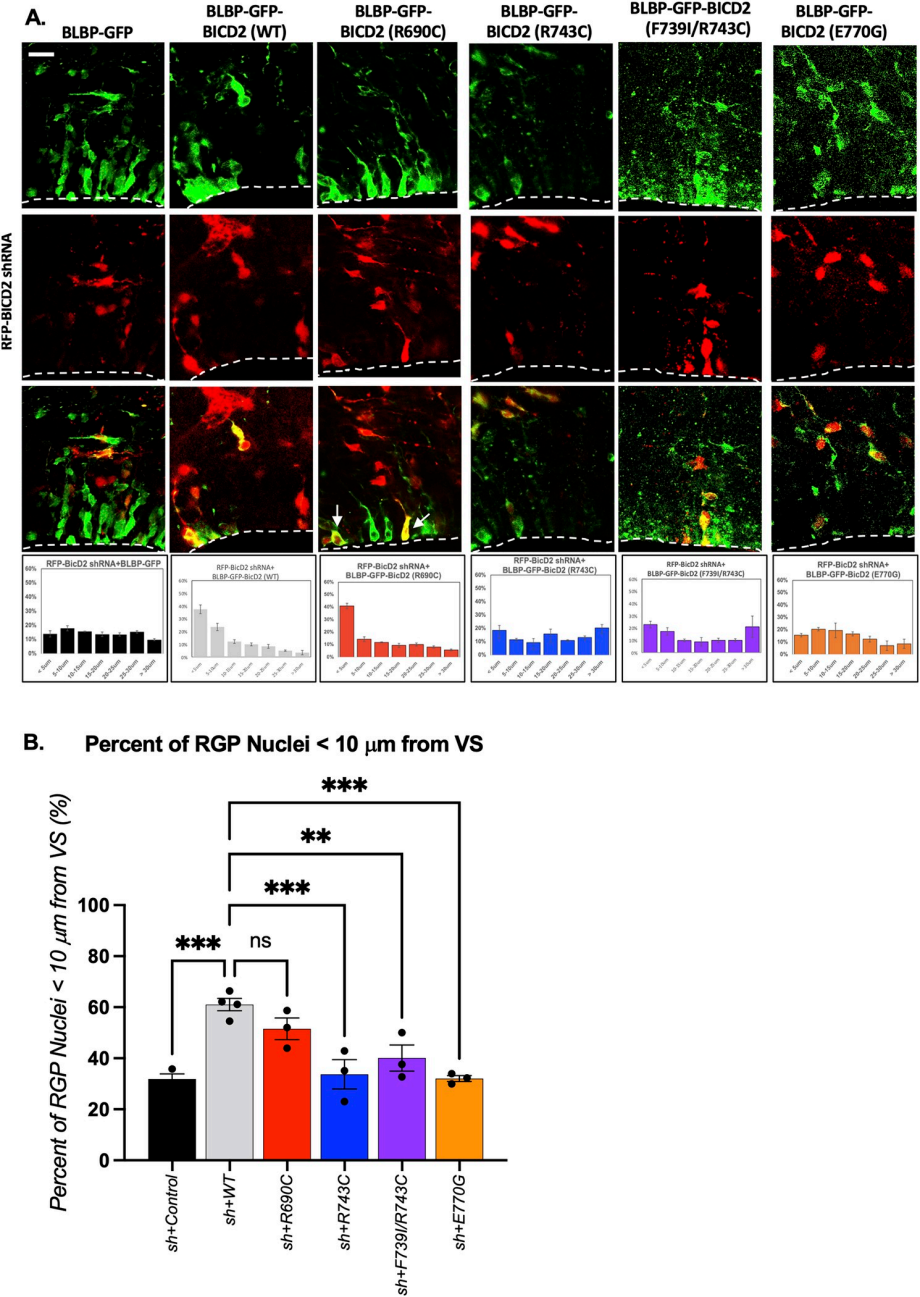
BICD2 mutations with defective RanBP2 binding interferes with apical nuclear migration in RGP cells, *in vivo*. E16 rat brain was *in utero* co-electroporated with an RFP-BICD2 shRNA [5] and GFP-tagged wildtype/mutant BICD2 cDNAs under control of the BLBP promoter for RGP specific expression to perform rescue experiments. Representative E20 rat brain slices are shown near the ventricular surface (VS) (*white* dashed line). (A) Top panels show green RGP cells expressing the GFP tagged BICD2 wild type or mutant constructs. The middle panels show RFP-BICD2 shRNA expressing cells followed by merged images of the GFP and RFP channels. The corresponding histograms of the measured distances from the VS to nuclei of co-electroporated cells are shown in the bottom panels. White arrows point to two RGP nuclei, expressing both the shRNA and the BicD2 constructs at ventricular surface. Detailed experimental design and the measuring method can be found in S4 Fig. Scale bar = 10μm. (B) Mean percent of the RGP nuclei within 10μm of the VS are plotted as bar graphs. All error bars are S.E.M from at least 3 different brains. Each black dot in panel B represents one embryonic rat brain. One-way ANOVA with Dunnette’s test was performed and the statistical significance is marked in black. (ns = not significant; ** p <0.01; *** p<0.001).

To test the physiological consequences of altered RanBP2-BICD2 binding seen with these mutations, we analyzed their effects in the embryonic rat brain. To focus on RGPs alone, we performed *in utero* electroporation of GFP-tagged full-length BICD2 cDNA constructs under the control of the RGP-specific promoter, BLBP [43] in E16 embryonic rat brains, which were collected at E19 or E20 for further processing and analysis. To test RanBP2-BICD2 mediated apical INM function, we determined the distance of wildtype vs. mutant BICD2-expressing RGP nuclei from the ventricular surface (VS) of developing brains (S4A and S4B Fig) as previously performed [5]. We note that this type of analysis has been previously used to show INM defects in multiple studies [43–45], validating the robustness of the analysis. Expression of the R690C mutant BICD2 cDNA had no effect on RGP nuclear distribution (S4D and S4E Fig). Moreover, the R690C mutation, which enhances the BICD2- RanBP2 interaction (Fig 3B and 3C), completely rescued BICD2-RNAi-associated defects in apical nuclear migration as evidenced by comparable number of RGP nuclei located within 10 μm from the VS as the wildtype control (Fig 4A and 4B). These results confirm that BICD2-RanBP2 mediated function is not affected by the R690C mutation. In contrast, the E770G mutant BICD2, which exhibited diminished RanBP2 binding, did not cause a change in RGP nuclear distribution when expressed alone (S4D and S4E Fig), but failed to rescue the apical INM defect induced by BICD2 RNAi (Fig 4A and 4B). In summary, the enhanced RanBP2-BICD2 interaction we observed for R690C appeared to restore RanBP2-BICD2 mediated dynein function in RGP cells in the developing brain. Conversely, the disrupted RanBP2-BICD2 interaction observed in cells expressing the BICD2 E770G mutant resulted in severe impairment of RanBP2-BICD2-mediated dynein function during apical nuclear migration in the RGPs (Fig 4A and 4B).

**Fig 5:**
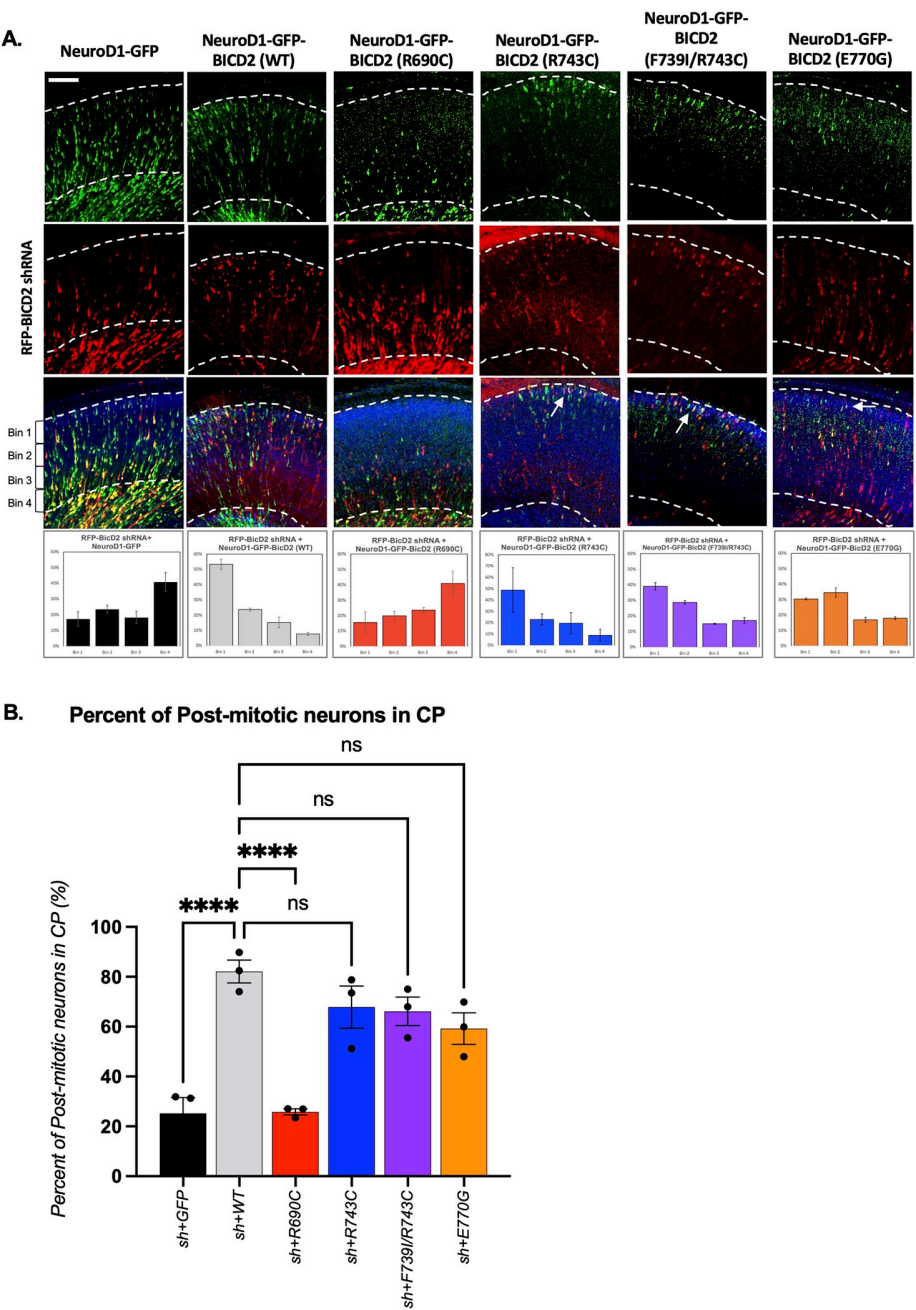
BICD2 R690C mutation causes a specific neuronal migration defect. E16 rat brain was *in* utero co-electroporated with GFP tagged wildtype/mutant BICD2 cDNAs under control of the NeuroD1 promoter for neuron specific expression and an RFP-BICD2 shRNA [5] to perform rescue experiments. E20 brain slices were imaged and the distribution of co-electroporated postmitotic neurons were analyzed. Representative cortical plate (CP) images are shown. (A) Top panels show post-mitotic neurons electroporated with wild type or mutant constructs. The middle panels show RFP-BICD2 shRNA expressing cells followed by merged images of the GFP and RFP channels. To quantify the postmitotic neuronal distribution in CP, distances from the pial surface (the upper white dashed line) to the cell were measured and the histograms are shown in the bottom panels. The relative bin size was calculated by assessing the cortical plate thickness for each brain slice, and then divided into four regions (Bin1, 2, 3, and 4). Scale bar = 100 μm. (B) Mean percent of the postmitotic neurons in the cortical plate. Error bars show the S.E.M from at least 3 different brains. Each black dot represents one brain. One-way ANOVA with Dunnette’s test was performed for statistical analysis. (ns = not significant; **** p<0.0001).

To test the consequence of the altered Nesprin-2-BICD2 binding, we engineered BICD2 cDNAs under control of the neuron-specific promoter, NeuroD1, to ensure expression in postmitotic neurons alone [43, 46]. Again, the constructs were introduced into E16 rat brains by *in utero* electroporation which were collected for analysis at E20. Expression of the mutant BICD2 constructs alone, again, caused no detectable phenotype (S5A and S5B Fig). However, cells expressing the NeuroD1- driven R690C mutant along with the BICD2 shRNA displayed severely impaired neuronal migration, as indicated by a 4-fold reduction in the percent of transfected cells in the cortical plate (Fig 5A and 5B). E770G, which enhances Nesprin-2-BICD2 binding (Fig 3D and 3E), fully rescued the RNAi-induced defect in the postmitotic neuronal migration to the pial surface as judged by comparable number of E770G positive neurons in the CP (Fig 5B).

Taken together, our data support the hypothesis that the impaired basal postmitotic neuronal migration associated with the BICD2 R690C mutation is caused by abnormally increased RanBP2-BICD2 binding, which, in turn, interfere with Nesprin-2-BICD2 binding, as a result of the binding competition between RanBP2 *vs*. Nesprin-2 for BICD2. Consequently, the R690C mutant resulted in impaired Nesprin-2-BICD2 mediated function. In contrast, the impaired apical INM seen for BICD2 E770G must be caused by a reduction in BICD2-RanBP2 binding affinity.

### A coiled-coil registry-shift- locking BICD2 mutant, F739I/R743C, preferentially binds to Nesprin-2

**Fig 6:**
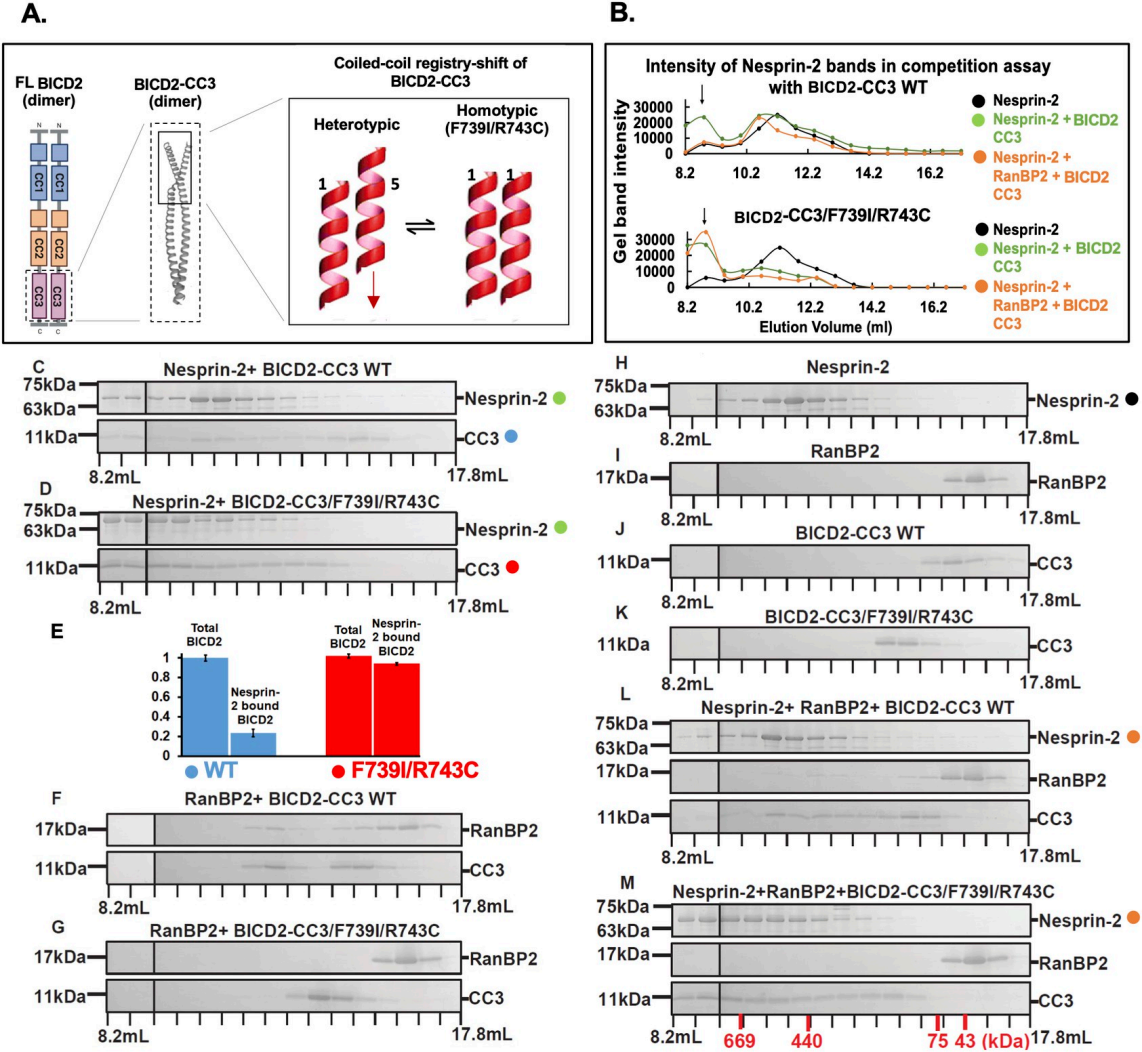
The registry-shift locked mutant F739I/R743C, preferentially binds to Nesprin-2. (A) Schematic depiction of the BICD2 dimer (left). CC3 is highlighted by a dashed box. The X-ray structure of human BICD2-CC3 in cartoon representation (middle; PDB ID 6OFP) is shown in the middle. The N-terminal half of the BICD2-CC3 dimer is highlighted by a box that is enlarged in the right panel. This region of BICD2 is likely undergoing a coiled-coil registry-shift from heterotypic to homotypic, which is depicted in a schematic manner. In the heterotypic registry, the two chains of the dimer are vertically displaced against each other by approximately one helical turn. In the homotypic coiled-coil registry, the chains are aligned at equal height [19]. (C,D and E-M) Purified Nesprin-2 fragment, RanBP2 fragment, and BICD2-CC3 were mixed in a 1:1:2 molar ratio (Nesprin-2: RanBP2: BICD2-CC3) and analyzed by size exclusion chromatography. SDS-PAGE analysis of the elution fractions and (B) intensity profiles of the gel bands are shown. (B) Intensity profiles of Nesprin-2 gel bands from the SDS-PAGEs shown in panels C, D, H, L and M (see color-coded circles). The Nesprin-2 elution peaks from the size exclusion chromatography assays with Nesprin-2, Nesprin-2 + BICD2-CC3 and Nesprin-2 + RanBP2 + BICD2-CC3 are compared for WT (top panel) and F739I/R743C mutant (bottom panel) BICD2-CC3. The arrows point to complex formation, indicated by a shift of the elution peak towards higher mass. (C-L) SDS-PAGE analyses of elution fractions are shown. Masses of molecular weight standards are shown on the left and elution volumes on the bottom. (C) Nesprin-2 + BICD2-CC3 WT. (D) Nesprin-2 + BICD2-CC3/F739I/R743C mutant. (E) The assays from (C, D) were repeated three times and each analyzed on a single SDS-PAGE together with 2 μg of WT BICD2 (see S6 Fig) in order to quantify the amounts of BICD2-CC3 WT (blue) and F739I/R743C (red) in the gel bands. The amounts were normalized to 1 for total BICD2. Total BICD2 (i.e. BICD2 in all fractions) is shown versus Nesprin-2-bound BICD2 (i.e. the BICD2 in the first nine fractions that co-elute with Nesprin-2 in S6 Fig). The error was calculated as standard deviation. (F) RanBP2 + BICD2-CC3 WT. (G) RanBP2 + BICD2-CC3 F739I/R743C. As controls, the individual proteins were also analyzed: (H) Nesprin-2 (I) RanBP2 (J) BICD2-CC3 (K) BICD2 CC3/F739I/R743C mutant. (L) Nesprin-2 + RanBP2 + BICD2-CC3 WT. (M) Nesprin-2 + RanBP2 + BICD2-CC3/F739I/R743C mutant. All experiments were repeated two times. The elution volumes of molar mass standards are indicated in red at the bottom of panel (M).

Among the structural features of the BICD2 molecule is the unusual ability of the CC3 region to form an α-helical coiled-coil structure in two distinct registry states, one in which the α-helices of the dimer are aligned at equal height (homotypic registry), and another, where the α-helices are vertically shifted by ~one helical turn in the N-terminal half of the coiled-coil (heterotypic registry). This behavior has been characterized in *Drosophila* BicD [16] and in mammalian BICD2 [18, 19, 47] (Fig 6A) and implicated in autoinhibition control of BICD2. Interestingly, the registry-locked F739I/R743C mutant, which likely locks the coiled-coil in a homotypic registry (the α-helices are aligned at equal height) (Fig 6A), was also found to diminish RanBP2 binding in pull-down assays, whereas the interaction with Rab6 was not affected [47]. However, the homotypic registry-locked mutant effect on Nesprin-2 binding has not been explored.

To test the relative effects of the registry-locking mutation F739I/R743C on RanBP2 *vs*. Nesprin-2 binding, we incubated the BICD2-CC3 (aa 711–800) F739I/R743C variant and the WT *in vitro* with our RanBP2 (aa 2148–2240) or Nesprin-2 (“N2G SR 52–56”) fragments and analyzed changes in the interaction behavior by size exclusion chromatography (Fig 6B–6M). Consistent with the previously reported effect of F739I/R743C on RanBP2 binding [47] (S7 Fig), the BICD2-CC3 F739I/R743C mutant fragment showed a nearly complete loss in its ability to interact with RanBP2 (Fig 6G and 6M). In contrast, the mutation markedly enhanced BICD2-Nesprin-2 (Fig 6B–6E), suggested by a more pronounced shift of the BICD2-CC3 elution peak towards higher mass observed for the F739I/R743C mutant than for the wildtype BICD2-CC3 in the BICD2-CC3/Nesprin-2 mixture (Fig 6B–6E). We also quantified the amount of total BICD2 versus Nesprin-2 bound BICD2 for the size exclusion experiment with the WT and the F739I/R743C mutant (Fig 6E, S6 Fig). While only approximately 23% of the BICD2-CC3 WT co-elutes with Nesprin-2, approximately 94% of the BICD2-CC3 F739I/R743C mutant co-elutes with Nesprin-2, supporting a marked increase of the binding affinity of the mutant towards Nesprin-2 compared to the WT.

In the competition assay with all three proteins (i.e., BICD2-CC3, RanBP2, and Nesprin-2), the F739I/R743C mutant also interacted more strongly with Nesprin-2 compared to the wildtype BICD2-CC3’s interaction with Nesprin-2 (Fig 6B). We repeated this analysis for the F739I single mutant, which likely locks the BICD2 coiled-coil registry in the same state as the F739I/R743C double mutant [19, 47]. The BICD2-CC3 F739I fragment again showed a nearly complete loss of RanBP2 binding while preserving the Nesprin-2 binding (S3 Fig). As expected, the effect was less pronounced in the F739I single mutant compared to the F739I/R743C double mutant (note that a similar competition assay could not be performed for the R743C mutant because of its additional adverse effect on the BICD2 coiled-coil stability, which was rescued in the double mutant [19]. Our data together indicate that each of two coiled-coil registry- locking mutations, F739I and F739I/R743C, which are presumed to lock the BICD2 coiled-coil in a homotypic registry, cause preferential binding to Nesprin-2, and both mutants have a markedly stronger binding affinity towards Nesprin-2 than the WT.

### *BICD2* registry-locking mutations also selectively interfere with INM in embryonic rat brain

To evaluate the physiological effects of the registry-locking mutations, we again used the embryonic rat brain system. We engineered mutant forms of full-length BICD2 cDNAs under the RGP-specific BLBP, or the neuron-specific NeuroD1 promoters. For these experiments, we focused on the two registry-locking mutations: the F739I/R743C and the R743C. For this *in vivo* study, the R743C mutant was used in view of its association with the SMA-LED2 [33] in addition to its effect on the α-helical coiled-coil registry [19]. Because the BICD2 R743C is believed to lock the α-helical coiled-coil in the same state as F739I/R743C [19], it is thus predicted to cause the same loss of RanBP2 binding as seen for the F739I/R743C mutant. We have previously shown that expression of WT BICD2 rescues BICD2-RanBP2 mediated INM functions in BICD2 knock-down cells [5]. Consistent with the loss of RanBP2 binding seen for the registry-locking mutations (Fig 6G and 6M), expression of the BICD2-R743C or -F739I/R743C mutants in the BICD2 knock-down cells *in vivo* was unable to rescue BICD2-RanBP2 mediated function, as evidenced by an ~40% reduction in the percent of RGP nuclei situated within 10 μm from the ventricular surface of the electroporated brains compared to the WT (Fig 4A and 4B). These data suggest that the disrupted RanBP2-BICD2 interaction associated with the BICD2 R743C and F739I/R743C mutations causes severe impairment of RanBP2-BICD2 mediated dynein function during apical nuclear migration in the RGP cells (Fig 4A and 4B).

In our biochemical analysis (Fig 6 and S3, S6 Figs), we also found the registry-locking mutants to enhance the BICD2-Nesprin-2 interaction (Fig 6B and 6E). Thus, the registry-locking mutants should reasonably be expected to restore normal postmitotic neuronal migration, given the Nesprin-2 mediated BICD2 role at the NE of postmitotic neurons. To test this hypothesis, we engineered BICD2 cDNAs bearing the R743C or F739I/R743C mutations, each under the control of NeuroD1 to ensure the postmitotic neuron-specific expression [43,46]. Consistent with our biochemical results, we observed no detectable effect of the BICD2 F739I/R743C or R743C mutants on post-mitotic neuronal migration as evidenced by normal numbers of mutant BICD2 mutant-expressing cells reaching the cortical plate *vs*. wildtype cells (S5A and S5B Fig). Moreover, expression of the mutant BICD2 fully rescued BICD2 RNAi-inhibited neuronal migration (Fig 5A and 5B) compared to the wildtype control BICD2. These results confirm the essential and mutually exclusive functions for the RanBP2- *vs*. Nesprin-2- BICD2 interactions in successive brain developmental stages. Also, the diminished RanBP2-BICD2 interactions observed for the R743C or F739I/R743C mutations again specifically blocked apical nuclear migration in the RGP cells without affecting basal migration of the postmitotic neurons.

## Discussion

We previously found that BICD2 interacts with two different NE-associated cargos in neuronal precursor cells in the developing brain, RanBP2 [5] and Nesprin-2 [3]. We now find these proteins to interact with a common region within the BICD2 C-terminal CC3 domain, although at apparently non-identical distinct sites. As a test for the dual role for this region in brain development, we expressed mutant forms of BICD2 that we determined by biochemical analysis to exhibit different affinities for Nesprin-2 *vs*. RanBP2. Together, the resulting data provide the most direct evidence to date that a single protein–BICD2 –contributes to the fundamental developmental shift from neural progenitor proliferation to neuronal migration and the assembly of the neocortex.

**Fig 7:**
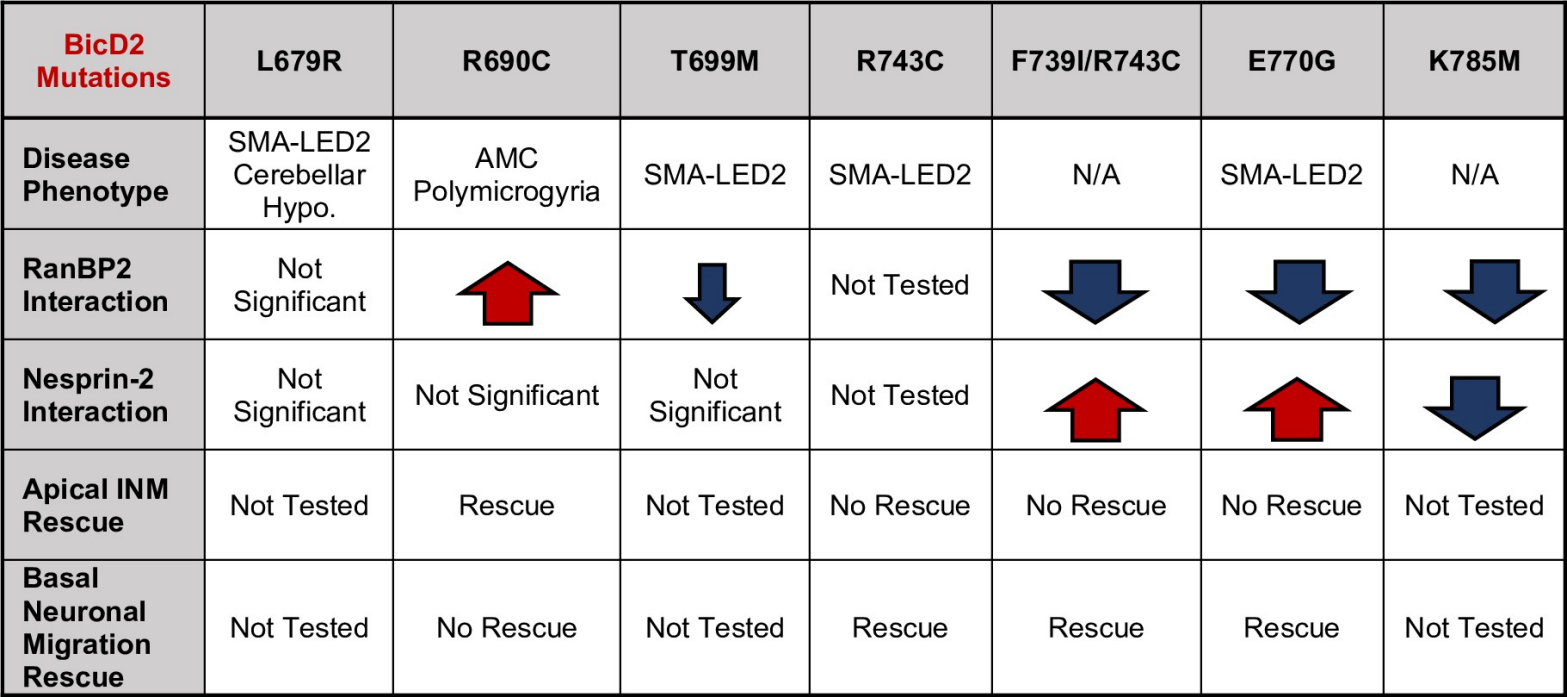
Summary of mutational effects.

Our study both advances our understanding of BICD2 cargo interactions and demonstrates the physiological consequences of perturbed cargo interactions *in vivo*. We find that two forms of BICD2 cargo -RanBP2 and Nesprin-2- compete for BICD2 binding. Moreover, we find that the BICD2 registry-locking F739I/R743C mutant enhances binding to Nesprin-2 while abolishing the RanBP2 interaction, a novel role for this remarkable form of protein conformational flexibility. Furthermore, the altered nuclear cargo interaction caused by the *BICD2* F739I/R743C mutation specifically impaired RanBP2-BICD2-mediated apical INM, without affecting postmitotic neuronal migration. We also found that two previously reported disease-causing BICD2 mutations—R694C (R690C) and E774G (E770G) in human BICD2 (mouse residue numbers in parenthesis) result in abnormal enhancement of RanBP2 or Nesprin-2 binding, respectively. These mutations caused nuclear migration defects in either the postmitotic neurons or the RGPs, thus confirming the exclusive roles for BICD2-RanBP2 and BICD2-Nesprin-2 complexes in the two successive brain developmental processes. In summary, our results validate our earlier hypothesis that BICD2 requires distinct nucleus-cargo interactions during successive stages of brain development (summarized in Fig 7), defects in which contribute to impaired nuclear migration in either of two fundamentally important but distinct cell types.

### BICD2 coiled-coil registry-shift as an underlying mechanism controlling RanBP2 *vs*. Nesprin-2 binding

A nucleoporin, RanBP2, and a nuclear transmembrane protein, Nesprin-2, are each exposed on the cytoplasmic surface of the NE and serve similar roles in recruiting dynein motors via BICD2 for nuclear migration. Each protein also makes direct contact with the CC3 domain of BICD2 but at somewhat different locations (Fig 1D). RanBP2 and Nesprin-2 can compete for BICD2 binding (Fig 2I–2K) despite their different interaction sites within the BICD2 molecule. And this mechanism is physiologically important as suggested by the physiological consequences of the *BICD2* mutations affecting RanBP2 *vs*. Nesprin-2 binding and in view of the low level of BICD2 expression *in vivo* [42].

We do not fully understand the molecular basis for the competition between RanBP2 and Nesprin-2 for BICD2 binding. However, we speculate that coiled-coil registry-shifting within CC3 might have a role in regulating the competition between the two NE proteins for the CC3 binding. The coiled-coil registry shift requires an ability of the α-helices to form a coiled-coil structure in more than one register. Upon the registry shift, a structural rearrangement of the amino acid side chains occurs [19], which would remodel the surface of the CC3 domain, thus allowing for a unique set of interactions. This registry-shifting mechanism has been implicated in controlling the autoinhibition of BICD2 [16, 47], which involves an intramolecular interaction of CC3 and CC1 domains, that could be weakened by a registry shift. Such interaction change of the CC3 domain with other CC3 interactors has seemed formally possible, but it has not been explored until this current study. We confirm that the registry-shift locking mutant (F739I/R743C) abolishes RanBP2 binding [47]. Moreover, this same registry-shifting mutation that is expected to lock the registry in one state, not only retained, but enhanced Nesprin-2 binding compared to the wildtype CC3 (Fig 6B). This result suggests that the CC3 registry shift can modulate the interaction of BICD2 with RanBP2 and/or Nesprin-2. And, the F739I/R743C mutation locks the CC3 dimer in the registry state disfavoring RanBP2, while favoring Nesprin-2 binding.

The relationship between the two apparent roles for BICD2 registry shift remains uncertain. One possibility, however, is that BICD2 interactions with its two forms of NE cargo adaptor–Nesprin-2 and RanBP2 are linked to autoinhibition. This might have the effect of modulating dynein motor activity differentially to the two forms of cargo. Clearly, more work will be needed to test this and other hypotheses, and for examining the role of registry shift in transport of other forms of dynein cargo.

### BICD2 registry-shift in autoinhibition *vs*. nuclear cargo interaction

Registry shift of the BICD2 CC3 has been previously implicated in the regulation of the BICD2 autoinhibition. The *Drosophila* homolog of a registry-locking mutation, F739I, was shown to activate BICD2 for dynein interaction even in the absence of a cargo [47]. Expression of this cargo-less but activated BICD2 could potentially result in sequestration of dynein pool in the cell, consequently depriving dynein motors from the cellular cargos in need of the dynein function, and it is conceivable that in the developing brain, such dynein deprivation may possibly result in impairment of both apical migration and postmitotic neuronal migration. However, it should be pointed out that the expression of the registry-shift mutant in the brain did not show such phenotypes in both RGPs (S4D and S4E Fig) and postmitotic neurons (S5A and S5B Fig), suggesting that the potential dynein deficiency caused by the registry-shift mutant expression was insignificant in the brain system. Instead, the impaired apical INM observed for R743C or F739I/R743C in BICD2 shRNA background was a direct consequence of the reduced RanBP2-BICD2 interaction caused by the mutation.

### R694C (R690C in mouse *Bicd2*) induced *gain-of-function* defect in RanBP2 binding and the brain phenotypes

R694C is a recurrent *BICD2* mutation that causes a severe form of congenital polymicrogyria [28] with impaired neuronal migration [39]. The underlying molecular defect induced by this mutation is currently unknown. Our work reveals that the mouse homolog of R694C (R690C) induces a postmitotic neuronal migration defect in the cerebral cortex, consistent with the disease phenotype and the previously published result [39]. And, here, we report further that the effect of the R690C is limited to postmitotic neurons, consistent with a specific role in this process but not in apical nuclear migration in RGP cells. As an underlying molecular defect for this mutational phenotype, we initially anticipated a decreased Nesprin-2 binding, which would result in loss of Nesprin-2-BICD2 mediated nuclear migration function. Surprisingly, we could not detect a significant change in binding affinity to exogenously expressed GFP-Nesprin-2 fragment, but we observed an abnormal ~2-fold increase in RanBP2 binding for the R690C mutant BICD2-CT in the GST pull-down assay (Fig 3B and 3C). The undetectable reduction in the GFP-Nesprin-2 fragment and BICD2 binding was likely caused by the excess amounts of exogenously produced GFP-Nesprin-2 in the cell lysate masking the phenotype. Considering our *in vitro* data demonstrating that RanBP2 *vs*. Nesprin-2 compete for binding to BICD2 CC3, the abnormally enhanced RanBP2-BICD2 interaction would have a negative effect on the Nesprin-2-BICD2 interaction, which would result in a defective postmitotic neuronal migration caused by deficient Nesprin-2-BICD2 interaction (Fig 5A and 5B).

In the developing brain, a change in BICD2-RanBP2 binding affinity naturally occurs as a function of cell cycle progression, and has already been proven to be a functionally important aspect in INM regulation [14]. During late G2, RanBP2 is phosphorylated by Cdk1, which in turn increases the affinity for BICD2 by 2~3 folds compared to the non-phosphorylated state [14]. Following G2, RanBP2 returns to the de-phospho state and its interaction with BICD2 is greatly reduced. Although the physiological significance of the weakened interaction of de-phospho-RanBP2 and BICD2 has not been studied, perhaps, it is precisely at this moment that the abnormally high affinity for RanBP2 in the BICD2 R690C mutant causes a problem, impeding the progression to the next developmental step.

### E774G (E770G in mouse *Bicd2*) induced a *gain-of-funct*ion defect in Nesprin-2 binding and the consequence in the brain

The E774G (E770G in mouse BICD2) mutation has been implicated in an autosomal dominant form of SMA without reported developmental brain abnormalities. In our biochemical assays, E770G showed reduced RanBP2 binding but a novel gain-of-function increase in Nesprin-2 binding. The reduced RanBP2 binding by the E770G mutation of BICD2 impaired apical INM. But, the effect of enhanced Nesprin-2–BICD2 interaction by E770G was not detectable in the postmitotic neuronal migration, which involves Nesprin-2-BICD2 mediated dynein function. Although we could not observe a discernible phenotype in the brain system, this gain-of-function defect might be more evident in another system such as in the multinucleated skeletal muscle cells, in which nuclei are evenly distributed near the cell periphery as a result of countervailing opposite-directed forces by kinesin-1 and cytoplasmic dynein at the nuclear membrane [48,49]. Here, Kinesin-1 and BICD2-dynein are localized to NE via Nesprin-2 and any net change in the forces would result in nuclei clustering, which has been linked to muscle diseases [50] including an SMA-LED2 case [32]. In this system, the abnormally enhanced Nesprin-2-BICD2 interaction by the E770G mutation would enhance the dynein force, shifting the balance to the minus direction, and consequently clustering the nuclei. This enhanced Nesprin-2-BICD2 mediated dynein function in the muscles might partially explain the neuromuscular disease phenotype associated with this mutation, but no muscle biopsy result was reported for the E770G mutation.

Together, our results show that the pathogenic BICD2 mutations R690C and E770G cause novel gain-of-function defects in BICD2-cargo interactions and result in preferential binding to either RanBP2 or Nesprin-2. These mutations caused nuclear migration defects in either the postmitotic neurons or the RGPs, thus confirming the exclusive roles for BICD2-RanBP2 and BICD2-Nesprin-2 mediated dynein recruitment in the two successive brain developmental processes, validating our hypothesis that BICD2 requires interactions with distinct proteins at the NE during successive stages of brain development. Furthermore, we provide new evidence that the sites of BICD2 mutations can be clearly reflected in the specific features of brain developmental abnormalities.

### Ethics statement

All the experiments were done in accordance with the animal welfare guidelines and the guidance of the Institutional Animal Care and Use Committee (IACUC) at Columbia University (Approved protocol: AC-AABF8552).

### *In utero* electroporation

Plasmids encoding for cDNA were injected into the developing brain at embryonic day 16 (E16) and electroporated as described previously [51]. In more detail, timed pregnant Sprague Dawley E16 rats were anesthetized with a ketamine xylazine cocktail administered intraperitoneally, and toe pinch was performed to ensure deep anesthesia. To avoid excessive heat loss during the surgical procedure, an external heating source was provided. For pain management, buprenorphine XR and bupivacaine were administered subcutaneously before the surgery. Abdominal cavity was opened and uterine horns were exposed and trans-illuminated for clear identification of the embryonic brain ventricles. For easy visualization of the DNA in the brain ventricular space, a non-toxic dye (Sigma, F7252) was added to the DNA before surgery and injected with a beveled glass needle. After injection, embryos were subjected to five electric impulses (50V, 50ms each, separated by 1s intervals) delivered by an electroporator (Harvard Apparatus ECM 830). The embryos were returned to the abdominal cavity and the wound was closed. Rats were monitored twice a day post-surgery.

### Immunohistochemistry

For embryonic brain harvesting, pregnant rats were re-anesthetized and the surgical wound was reopened 3 or 4 days post injection, at E19 or E20, respectively to expose the uterus.

For fixed imaging, embryonic E19 or E20 rat brains were harvested and immersed in PBS with 4% PFA overnight. They were then embedded in 4% agarose (Sigma, A9539) and sliced using a vibratome (Leica, VT 1200S) in 100μm slices. After blocking in 5% normal donkey serum (Sigma, D9663) in PBS- 0.5% Triton X-100 for 1 hour at room temperature, slices were incubated with primary antibodies in PBS-5% Triton X-100, overnight at 4°C. Secondary antibodies (1:500) and DAPI (4’,6-diamidino-2-phenylindole, Thermo Scientific, 62248, 1:10.000 dilution) were diluted in PBS and incubated for 2 h at room temperature. Slices were mounted with Aqua-Poly mounting media (Polysciences, 18606).

### RNAi and cDNA expressing constructs

For the *in utero* electroporation experiments in the embryonic brain, BLBP- and NeuroD1- promoters were used to control cell type specific expression of the BicD2 constructs. The N-terminal GFP tagged full-length mouse Bicd2 isoform 2 (Accession: NP_084067.1) was synthesized (Synbio Technologies, New Jersey, USA) and inserted into BLBP-cre-GFP [43] between NcoI and Xbal sites, thus replacing cre-GFP with GFP-BICD2 to create BLBP-GFP-BICD2. Similarly, the same GFP-BICD2 was inserted into pNeuroD1-cre (gift from Dr. Carlos Cardoso, Institut de Neurobiologie de la Méditerranée) between EcoRI and NotI restriction sites to create NeuroD1-GFP-BICD2. RFP-BICD2 shRNA is from [5].

The constructs for bacterial expression were GST-His^6^-BICD2CT [14] and GST-BICD2 binding RanBP2 fragment (gift from Dr. Anna Akhmanova) [7]. GFP-N2G SR (Referred to as GFP-Nespin2 here) [3] was expressed in HeLaM cells.

All the missense mutations used in this study were introduced to the corresponding wild type constructs by PCR mutagenesis using the following primers:

**Table:** 

**Primer Name**	**Sequence (5’-3’)**
BicD2_L679R_FW	GAGATCCTCAAGCTGAAGTCCCTGCGGAGTACCAAGCGGGAGCAGATC
BicD2_L679R_REV	GATCTGCTCCCGCTTGGTACTCCGCAGGGACTTCAGCTTGAGGATCTC
BicD2_R690C_FW	AAGCGGGAGCAGATCACCACACTGTGCACCGTGCTCAAGGCCAACAAG
BicD2_R690C_REV	CTTGTTGGCCTTGAGCACGGTGCACAGTGTGGTGATCTGCTCCCGCTT
BicD2_T699M_FW	TGCTCAAGGCCAACAAGCAGATGGCTGAGGTGGC
BicD2_T699M_REV	ACGAGTTCCGGTTGTTCGTCTACCGACTCCACCG
BicD2_F739I_FW	GCAGGGAGGAGATGGTGGCTGCGTC
BicD2_F739I_REV	GACGCAGCCACCATCTCCTCCCTGC
BicD2_R743C_FW	GGCAAACATGGCACACAGGGAGGAGAAGG
BicD2_R743C_REV	CCTTCTCCTCCCTGTGTGCCATGTTTGCC
BicD2_E770G_FW	GCAGCTGCCGAGGACGGGAAGAAGACCCTTAAC
BicD2_E770G_REV	GTTAAGGGTCTTCTTCCCGTCCTCGGCAGCTGC
BicD2_K785M_FW	CATGGCCATCCAGCAGATGCTGGCGCTCACCCAG
BicD2_K785M_REV	CTGGGTGAGCGCCAGCATCTGCTGGATGGCCATG

#### Analytical size exclusion chromatography and pull-down assays with recombinant proteins

**Protein expression and purification:** Recombinant proteins (RanBP2, Nesprin-2, BICD2 fragments and mutants) for analytical size exclusion chromatography and pulldown assays were expressed and purified as described in [41, 47] with the following modifications. In brief, all expression constructs were obtained from commercial gene synthesis and codon optimized for expression in *E*. *coli* (Genscript). For sequences of *Ms* BICD2-CC3 (aa 711–800) and *Hs* RanBP2 (aa 2148–2240) see [40]. For the sequence of *Ms* Nesprin-2, see S1A Fig. The *E*. *coli* LOBSTR-BL21(DE3)-RIL strain was used for expression of BICD2 fragments. The expression plasmid for the mouse Nesprin-2 fragment (SR52-AD in the pGEX-6P-1 vector [21]) was obtained from Gregg G Gundersen. Nesprin-2 was expressed in the *E*. *coli* Rosetta 2(DE3)-pLysS strain, which was grown at 37°C until an OD_600nm_ of 0.6 was reached, then induced with 0.2 mM IPTG and incubated for 16 h at 16°C. GST-tagged Nesprin-2 was purified by glutathione affinity chromatography using the protocol described in [52]. Human RanBP2 (aa 2148–2240) was expressed as GST-fusion protein from the pGEX-6P-1 vector. RanBP2 was purified by glutathione affinity chromatography and eluted by proteolytic cleavage of the GST-tag with the PreScission protease (GE Healthcare). *Ms* BICD2-CC3 contained aa 711–800 which is identical in protein sequence to the human homolog *Hs* BICD2-CTD (aa 715–804). *Ms* BICD2 aa 750–800 and aa 630–800 were cloned into the pet28a vector with the NdeI and XhoI restriction sites. BICD2 fragments were purified by Ni-NTA affinity chromatography. For the assay shown in Fig 2, the His_6_-tag of BICD2-CC3 was removed by proteolytic cleavage by thrombin, followed by a second purification step of affinity chromatography. BICD2-CC3 (for Fig 2) and RanBP2 (for Figs 2, 6 and S3) were further purified by size exclusion chromatography as described in Cui et al., 2019 [41]. The gel filtration buffer was composed of 20 mM HEPES pH 7.5, 150 mM NaCl, 0.5 mM TCEP.

**Analytical size exclusion chromatography** was performed on the equilibrated Superdex 200 Increase 10/300 GL column (GE Healthcare). The gel filtration buffer contained 20 mM HEPES pH 7.5, 150 mM NaCl, 0.5 mM TCEP. For the assay in Fig 2, 1 mM MgCl_2_ was added to the buffer. Proteins were mixed in equimolar ratio, unless otherwise stated, and then filtered (pore size 0.02 μm) and centrifuged at 21700 x g for 25 minutes at 4°C. A sample volume of 550 μL was injected onto the column using a 500 μL sample loop. Elution fractions were analyzed by SDS-PAGE using 16% acrylamide gels and stained with Coomassie Blue. Gel band intensities were quantified with the software ImageJ [53]. A background subtraction with a blank gel band was performed. BICD2 and Nesprin-2 amounts were calculated by using defined μg amounts of BICD2 fragments that were analyzed on the same gel as standards. The obtained amounts were divided by the molar masses of the fragments to convert them to molar concentrations. For Fig 2, 1.3 mg Nesprin-2, 0.2 mg RanBP2 and 0.2 mg BICD2-CC3 were used. For Fig 6, and S3 and S6 Figs 1.3 mg of Nesprin-2, 0.2 mg of RanBP2 and 0.4 mg of BICD2-CC3 were used, which is a molar ratio of 1:1:2. The column was calibrated with the gel filtration calibration kits from Cytiva.

**GST-pull down assays** of GST-tagged Nesprin-2, GST-tagged RanBP2 and His_6_-tagged BICD2 fragments were performed as described in Cui et al., 2020 [47]. For these assays, BICD2 fragments were purified by a single affinity chromatography step from 1L of cell culture, as described in Cui et al., 2020 [47]. GST-RanBP2, GST-Nesprin-2 and GST (which was expressed from the pGEX6p1 vector) were purified from 0.5L of cell culture. For the GST-pull-downs, RanBP2 and Nesprin-2 were purified by glutathione Sepharose as described in Cui et al., 2018 [52] but not eluted. Purified BICD2 was added to the column, the column was washed, eluted and the eluate was analyzed as described in Cui et al., 2020 [47]. Gel band intensities were quantified as described above for analytical size exclusion chromatography.

### Cell culture and transfection

HeLaM cells (gift from Dr. Viki Allan, University of Manchester) were cultured in DMEM supplemented with 10% FBS at 37°C with 5% CO_2_. Transfections was performed with Effectene (Qiagen). Two days after the transfection, cells were harvested on ice with RIPA buffer (pH = 7.4, 50mM Tris-HCL, 125mM NaCl, 1mM EGTA, 0.5% NP-40) containing 1mM DTT and a protease inhibitor cocktail (Sigma, P8340).

### Protein purification, GST Pull-downs and Western blots

Bacterially expressed recombinant proteins with GST tags were incubated with Glutathione magnetic beads (Thermo Scientific, 78601) for 30min at 4°C to allow binding of the protein to the beads. Subsequently, beads were incubated with either untransfected or GFP-N2G SR (referred to as GFP-Nesprin-2) expressing HeLaM lysate for 3 hrs. at 4°C.Beads were pelleted and washed 3 times with the same reaction before elution. Eluates were retrieved by boiling the beads in laemelli buffer. Then the eluates were loaded on a polyacrylamide gel and transferred to a polyvinylidene difluoride membrane. The membrane was blocked in PBS with 5% milk, incubated with primary antibodies diluted in either PBS with 0.5% Tween or PBS with 1% milk, washed and incubated with secondary LI-COR antibodies in PBS. Imaging of the blots was carried out using an LI-COR.

### Antibodies

Antibodies used for immunofluorescence in brain slices include anti-GFP (1:400, Invitrogen, A11122), anti-RFP (1:400, MBL, M155-3) and donkey fluorophore-conjugated secondary antibodies (Jackson Labs, 1:500).

Antibodies used for western blotting include anti GST (1:3000, Santa Cruz, sc-53909), anti GFP (1:3000, Invitrogen, A11122), and anti-RanBP2 (1:1000, Abcam, ab64276). To develop in a LI-COR system, fluorescent secondary antibodies (1:10,000) were acquired from Invitrogen and Rockland.

#### Imaging and analysis

All the images were collected with an IX80 laser scanning confocal microscope (Olympus FV100 Spectral Confocal System). Brain sections were imaged using a 60x 1.42 N.A. oil objective or a 10x 0.40 N.A. air objective. All images were analyzed using ImageJ software (NIH, Bethesda, MD, USA).

## Supporting information

S1 Fig (A) Sequence of the “GST-Nesprin-2” fragment [21]. (B) Representative dataset (of three total) for the quantification shown in Fig 1D. Distinct amounts of BICD2 fragments (aa 630–800, 711–800 and 750–800) were analyzed on SDS-PAGE. An SDS-PAGE of the elution fractions of the GST-pulldown assays of Nesprin-2 with the three BICD2 fragments is shown. The distinct amounts of BICD2 fragments were analyzed on the same gel as the corresponding pull-down assays with the same fragments.
(TIF)

S2 Fig Mouse BICD2 and Human BICD2 are highly conserved within the C- terminal domain. Sequence alignment of mouse BicD2 isoform 2 (Accession: NP_084067.1) and human BICD2 isoform 1 (Accession: NP_001003800.**1).** The gray highlighted region corresponds to the “BicD2 CT” (aa 630–820) in Fig 3, which is identical in mouse (aa 630–820) and human BICD2 (aa 634–824). The point mutations used in this study are marked in red. Residue of mouse BICD2-CT corresponds to residue i+4 of the human BICD2-CT.
(TIF)

S3 Fig The BicD2-CC3 F739I mutant preferentially binds to Nesprin-2. (A-J) Purified Nesprin-2 fragment, RanPB2 fragment, and BicD2-CC3 were mixed in 1:1:2 molar ratio (Nesprin-2: RanBP2: BicD2-CC3) and analyzed by size exclusion chromatography. An SDS-PAGE of the elution fractions is shown. Masses of molecular weight standards are shown on the left and elution volumes on the bottom. (A) Nesprin-2 + BicD2-CC3 WT. (B) Nesprin-2 + BicD2-CC3/F739I mutant. (C) RanBP2 + BicD2-CC3 WT. (D) RanBP2 + BicD2-CC3 F739I. (E-H) As controls, the individual proteins were also analyzed: I) Nesprin-2 (F) RanBP2 (G) BicD2-CC3 (H) BicD2 CC3/F739I mutant. (I) Nesprin-2 + RanBP2 + BicD2-CC3 WT. (J) Nesprin-2 + RanBP2 + BicD2-CC3/F739I mutant. All experiments were repeated three times. Note that panels (A,C, E-G and I) were reproduced from Fig 6. The elution volumes of molar mass standards are indicated in red at the bottom of panel J.
(TIF)

S4 Fig (A) RGP Nucleus to ventricular surface distance measurement method. A coronal section of an E20 rat brain stained with anti-NeuN (red) for neurons and anti-Pax6 (green) for RGPs. Different sections of the brain are marked: Cortical Plate, IZ (intermediate zone), SVZ (Sub-ventricular zone), and VZ (ventricular zone). RGP nuclei reside within the VZ. (B) A representative image of an RGP near the ventricular surface is shown. Distance between the VS to the bottom of a RGP nucleus is measured (white dashed line) then (C) plotted as histogram (percent of cells vs. distance) as shown in the bottom panels of Fig 4A and S4D Fig. (D) GFP-tagged wild type or mutant BICD2 cDNAs were *in utero* electroporated and representative E20 rat brain slices are shown as in Fig 4. The top panels show green RGP cells expressing the GFP tagged BICD2 wild type or mutant constructs. The bottom panels are corresponding histogram (bin size 5 μm) of the nucleus to the VS distances as described above. Scale bar = 10μm. (E) Mean percent of the RGP nuclei within 10μm of the VS are plotted as bar graphs. All error bars are S.E.M from at least 3 different brains. Each black dot in panel E represents one embryonic rat brain. One-way ANOVA with the *post hoc* Dunnette’s multiple comparison test was performed against the wild type condition. BicD2 WT expression alone showed slight increase in the percent of RGP nuclei in the 10 μm from VS compared to the GFP alone control. The statistical significance is marked in black. (ns = not significant; * p<0.05).
(TIF)

S5 Fig Expression of BICD2 constructs show normal cortical migration. **(A)** GFP tagged BicD2 constructs under control of the NeuroD1 promoter were *in utero* electroporated at E16 and harvested at E20. The top panels show representative cortical plate images and the bottom panels show the percent of GFP positive neurons in each bin (marked in white) of the cortical plate. (B) Percent of GFP positive cells in the cortical plate are shown. Error bars are S.E.M.. Each black dot in (A) represents one brain. Ordinary one-way ANOVA with the *post hoc* Dunnette’s test against BICD2 WT was performed for statistical analysis (ns = not significant). Scale bar = 100 μm.
(TIF)

S6 Fig The assays from Fig 6C and 6D were repeated three times and each analyzed on a single SDS-PAGE together with 2 μg of WT BICD2 (first lane; labeled SD) in order to quantify the amounts of BICD2-CC3 WT (blue) and F739I/R743C (red) in the gel bands. A representative dataset is shown. The quantification is shown in Fig 6E.
(TIF)

S7 Fig BICD2 WT and F739I/R743C subcellular localization in G2 phase HeLa cells. Full-length **GFP tagged** BICD2 WT and the F739I/R743C variant were transiently transfected into HeLa cells that were fixed and immunostained with antibodies against GFP, the G2 phase marker Cyclin B1 and the dynein heavy chain and representative images of WT and F739I/R743C expressing G2 HeLa cells are shown in panels (A) and (B), respectively. While BICD2 WT clearly localized to the nuclear envelope (NE) (A), the F739I/R743C variant showed more diffuse staining at the nuclear envelope in Cyclin B1 positive cells (B). Consistently, the dynein heavy chain staining shows clear NE localization in the WT, but more diffused in the F739I/R743C. Scale bar = 10 μm. **Methods:** Effectene reagent (QIAGEN) was used for transfection of GFP-BICD2 plasmids [14] in HeLa cells as described in [14]. The medium was replaced after 6 h. Cells were incubated for 1 hr. in Nocodazole (10 μM) prior to fixation, which was performed 24 h post-transfection. For immunostaining, cells were washed in PBS, fixed in -20°C methanol for 10 min, washed in PBS and incubated for 1h with donkey serum in PBS. Immunostaining was performed for 2 h at 37°C with 1:200 dilutions of the following antibodies in blocking solution: Chicken polyclonal antibody against GFP (Millipore AB 16–901), rabbit dynein heavy chain polyclonal #46 antibody [54] and mouse Cyclin B1 antibody (Santa Cruz SC-245). Cover slips were washed with PBS and incubated with 1:200 dilutions in blocking solution of donkey fluorophore-conjugated secondary antibodies (Alexa Fluor 488, Cy3, Alexa Fluor 647, Jackson Immuno Research) for 1 h at room temperature. Cover slips were washed and mounted using AquaPoly/Mount (Polysciences Inc).
(TIF)

S1 Data “Raw Data and Statistics.zip” includes source data and statistics to Figs 1D, 3C, 3E, 4B, S4E, 5B, and S5B. (ZIP)
