# Supplementary figures and images for: Role of Nesprin-2 and RanBP2 in BICD2-associated brain developmental disorders
Source: PLoS Genet. 2023 Mar 17;19(3):e1010642. doi: 10.1371/journal.pgen.1010642 (PMC10022797; doi:10.1371/journal.pgen.1010642)

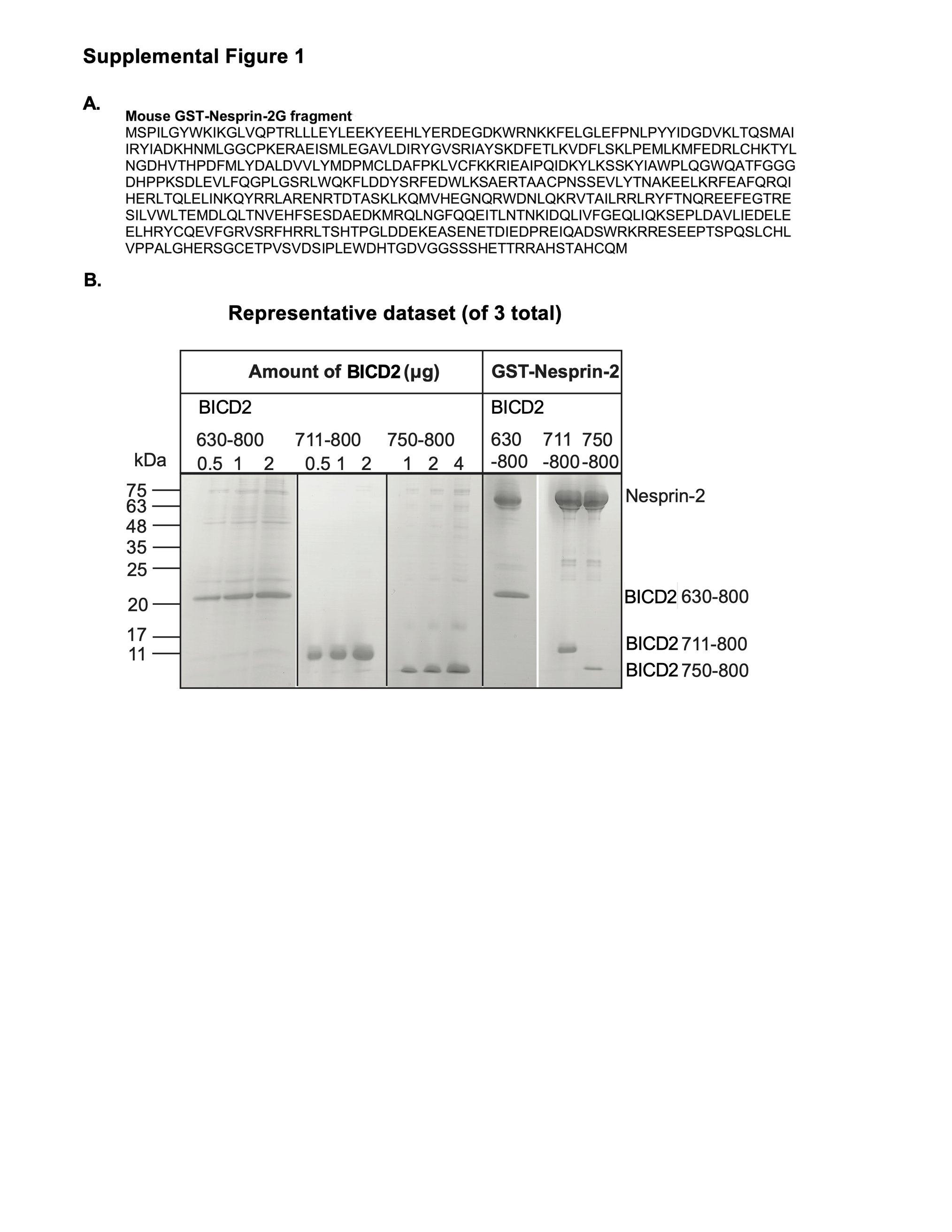

Supplement: S1 Fig — (A) Sequence of the “GST-Nesprin-2” fragment [21]. (B) Representative dataset (of three total) for the quantification shown in Fig 1D. Distinct amounts of BICD2 fragments (aa 630–800, 711–800 and 750–800) were analyzed on SDS-PAGE. An SDS-PAGE of the elution fractions of the GST-pulldown assays of Nesprin-2 with the three BICD2 fragments is shown. The distinct amounts of BICD2 fragments were analyzed on the same gel as the corresponding pull-down assays with the same fragments. (TIF) [file pgen.1010642.s001.tif]

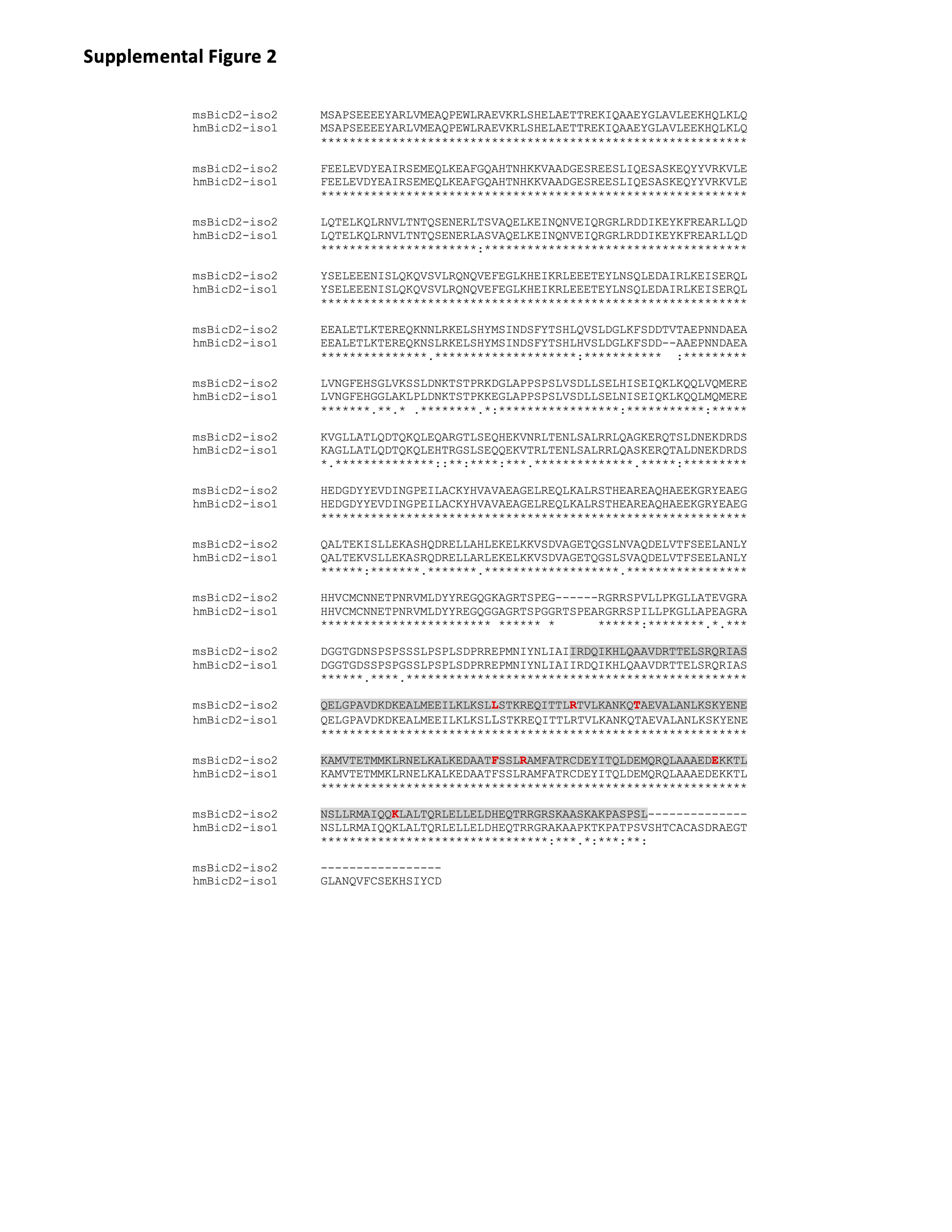

Supplement: S2 Fig — Sequence alignment of mouse BicD2 isoform 2 (Accession: NP_084067.1) and human BICD2 isoform 1 (Accession: NP_001003800.1). The gray highlighted region corresponds to the “BicD2 CT” (aa 630–820) in Fig 3, which is identical in mouse (aa 630–820) and human BICD2 (aa 634–824). The point mutations used in this study are marked in red. Residue of mouse BICD2-CT corresponds to residue i+4 of the human BICD2-CT. (TIF) [file pgen.1010642.s002.tif]

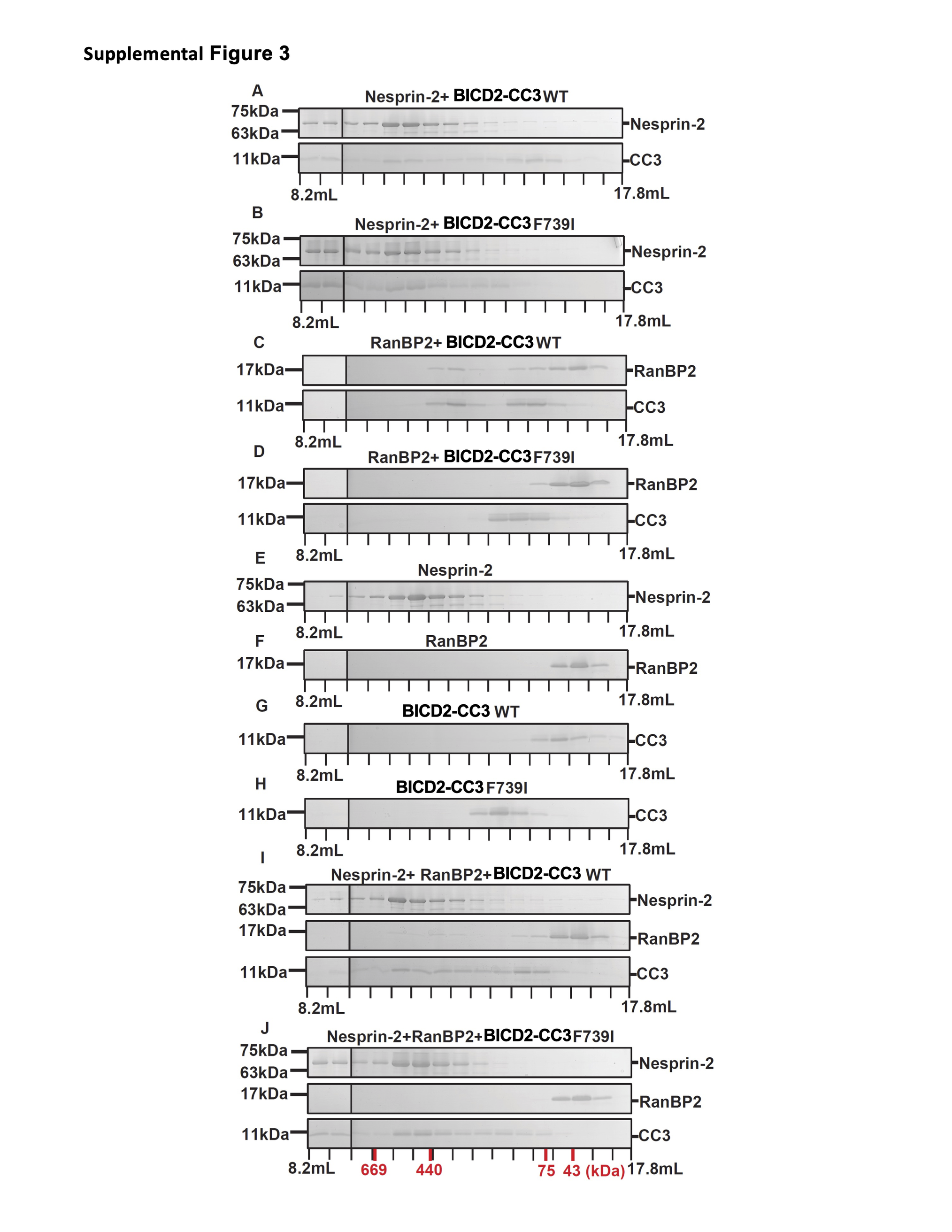

Supplement: S3 Fig — (A-J) Purified Nesprin-2 fragment, RanPB2 fragment, and BicD2-CC3 were mixed in 1:1:2 molar ratio (Nesprin-2: RanBP2: BicD2-CC3) and analyzed by size exclusion chromatography. An SDS-PAGE of the elution fractions is shown. Masses of molecular weight standards are shown on the left and elution volumes on the bottom. (A) Nesprin-2 + BicD2-CC3 WT. (B) Nesprin-2 + BicD2-CC3/F739I mutant. (C) RanBP2 + BicD2-CC3 WT. (D) RanBP2 + BicD2-CC3 F739I. (E-H) As controls, the individual proteins were also analyzed: I) Nesprin-2 (F) RanBP2 (G) BicD2-CC3 (H) BicD2 CC3/F739I mutant. (I) Nesprin-2 + RanBP2 + BicD2-CC3 WT. (J) Nesprin-2 + RanBP2 + BicD2-CC3/F739I mutant. All experiments were repeated three times. Note that panels (A,C, E-G and I) were reproduced from Fig 6. The elution volumes of molar mass standards are indicated in red at the bottom of panel J. (TIF) [file pgen.1010642.s003.tif]

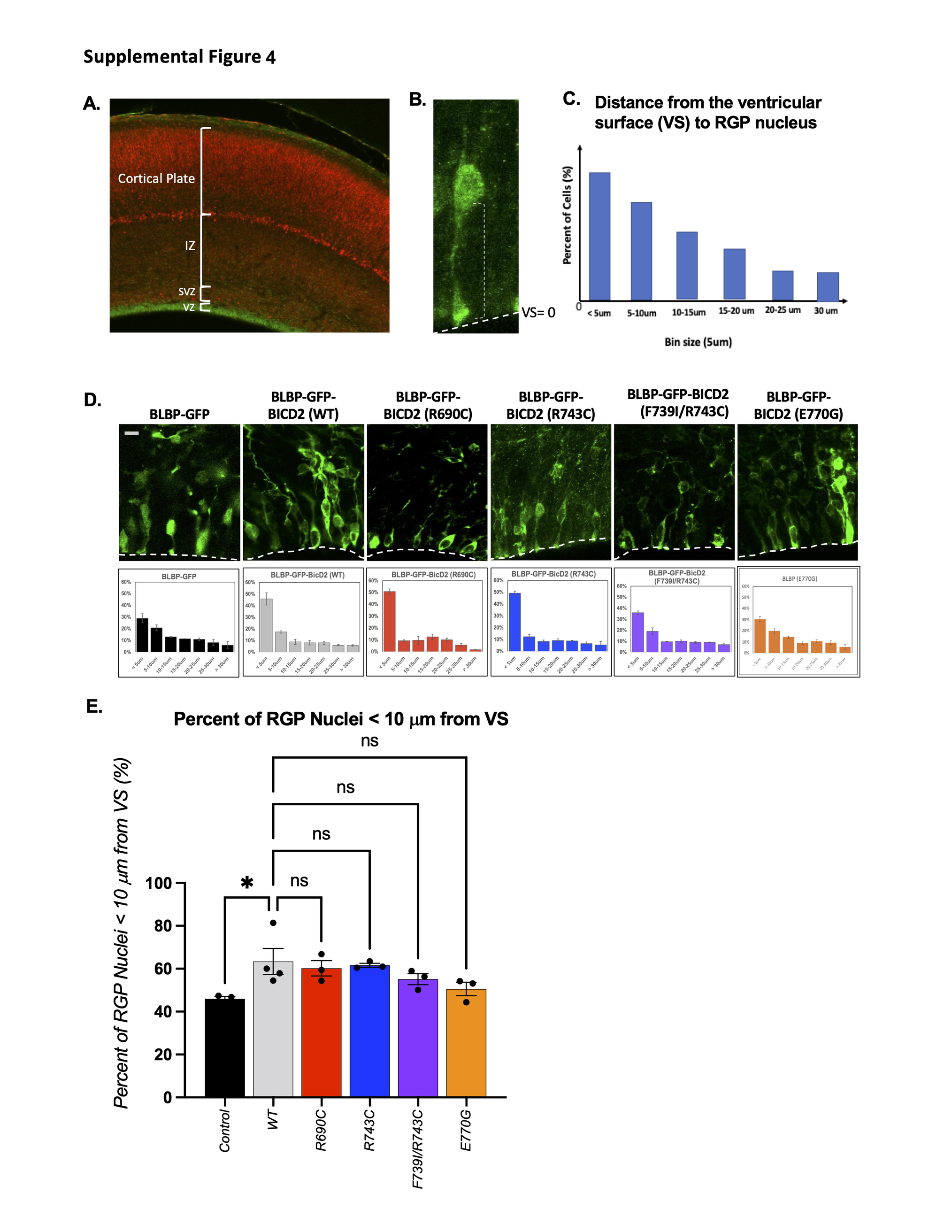

Supplement: S4 Fig — (A) RGP Nucleus to ventricular surface distance measurement method. A coronal section of an E20 rat brain stained with anti-NeuN (red) for neurons and anti-Pax6 (green) for RGPs. Different sections of the brain are marked: Cortical Plate, IZ (intermediate zone), SVZ (Sub-ventricular zone), and VZ (ventricular zone). RGP nuclei reside within the VZ. (B) A representative image of an RGP near the ventricular surface is shown. Distance between the VS to the bottom of a RGP nucleus is measured (white dashed line) then (C) plotted as histogram (percent of cells vs. distance) as shown in the bottom panels of Fig 4A and S4D Fig. (D) GFP-tagged wild type or mutant BICD2 cDNAs were in utero electroporated and representative E20 rat brain slices are shown as in Fig 4. The top panels show green RGP cells expressing the GFP tagged BICD2 wild type or mutant constructs. The bottom panels are corresponding histogram (bin size 5 μm) of the nucleus to the VS distances as described above. Scale bar = 10μm. (E) Mean percent of the RGP nuclei within 10μm of the VS are plotted as bar graphs. All error bars are S.E.M from at least 3 different brains. Each black dot in panel E represents one embryonic rat brain. One-way ANOVA with the post hoc Dunnette’s multiple comparison test was performed against the wild type condition. BicD2 WT expression alone showed slight increase in the percent of RGP nuclei in the 10 μm from VS compared to the GFP alone control. The statistical significance is marked in black. (ns = not significant; * p<0.05). (TIF) [file pgen.1010642.s004.tif]

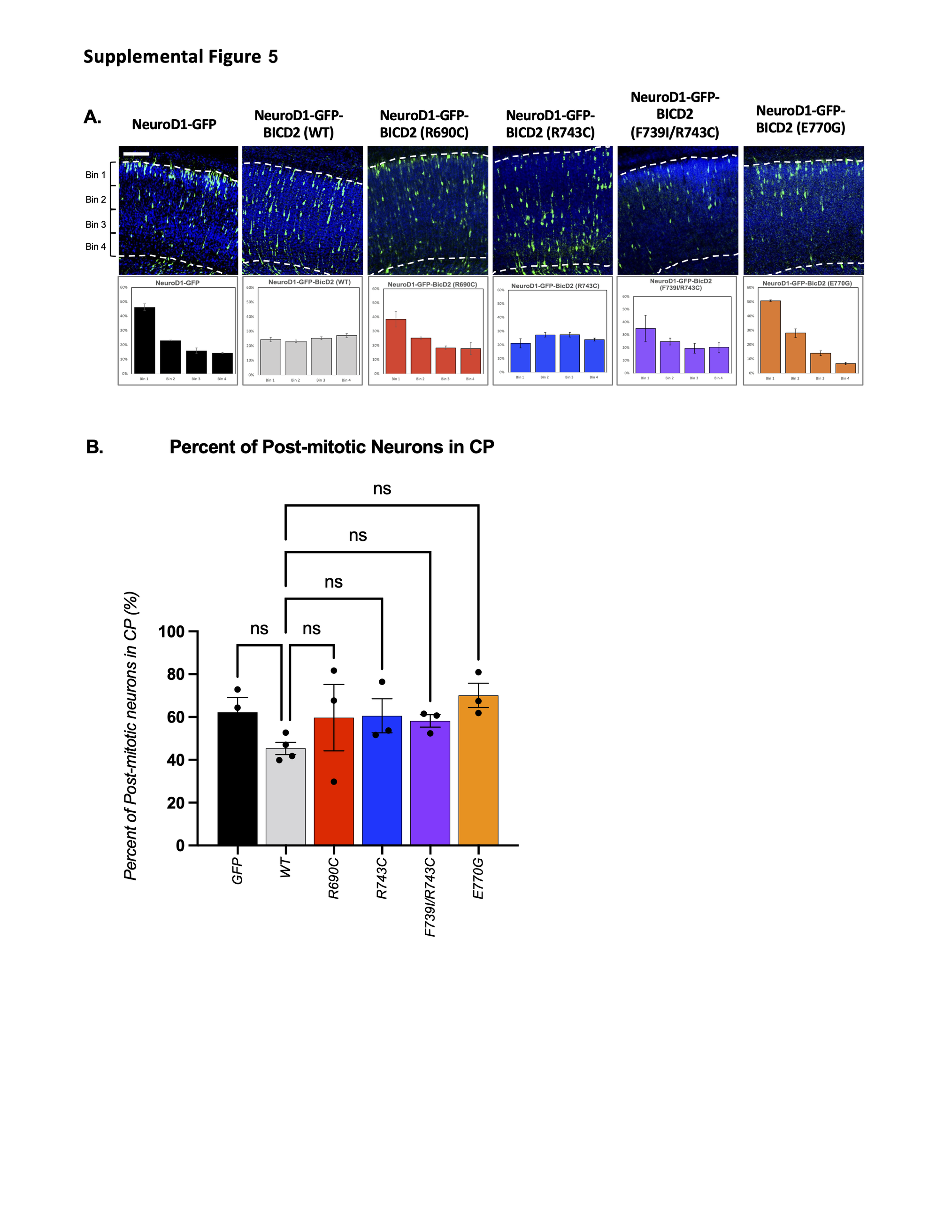

Supplement: S5 Fig — (A) GFP tagged BicD2 constructs under control of the NeuroD1 promoter were in utero electroporated at E16 and harvested at E20. The top panels show representative cortical plate images and the bottom panels show the percent of GFP positive neurons in each bin (marked in white) of the cortical plate. (B) Percent of GFP positive cells in the cortical plate are shown. Error bars are S.E.M.. Each black dot in (A) represents one brain. Ordinary one-way ANOVA with the post hoc Dunnette’s test against BICD2 WT was performed for statistical analysis (ns = not significant). Scale bar = 100 μm. (TIF) [file pgen.1010642.s005.tif]

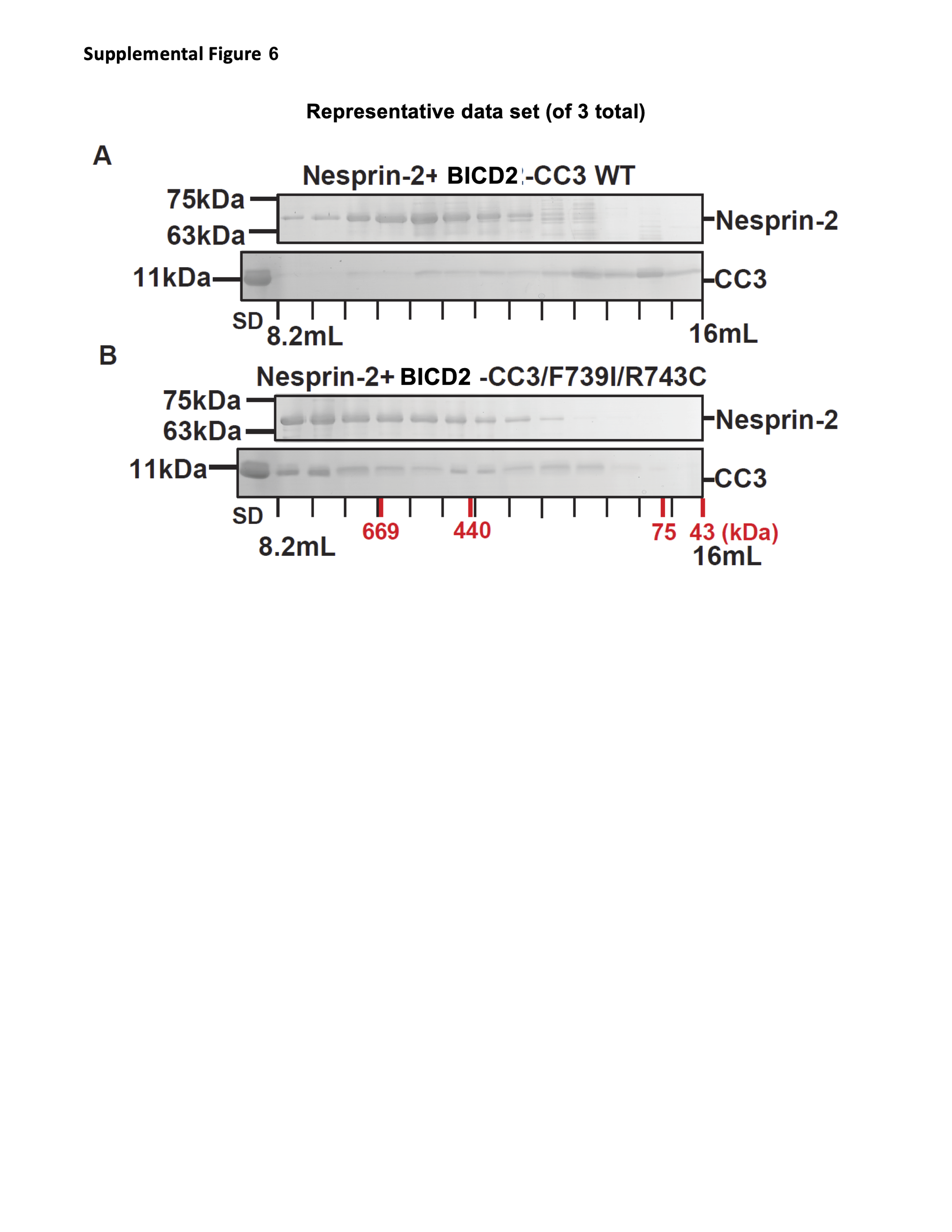

Supplement: S6 Fig — The assays from Fig 6C and 6D were repeated three times and each analyzed on a single SDS-PAGE together with 2 μg of WT BICD2 (first lane; labeled SD) in order to quantify the amounts of BICD2-CC3 WT (blue) and F739I/R743C (red) in the gel bands. A representative dataset is shown. The quantification is shown in Fig 6E. (TIF) [file pgen.1010642.s006.tif]

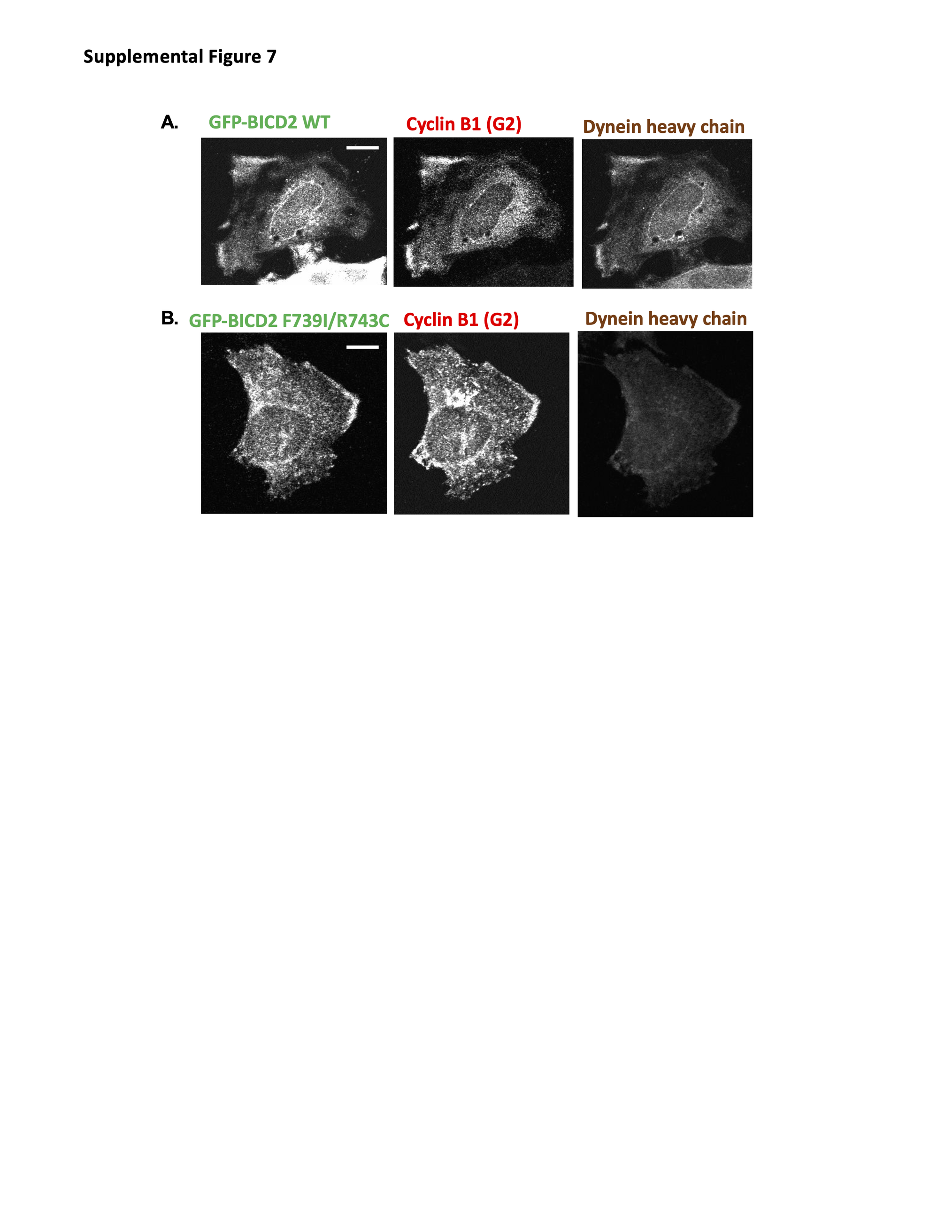

Supplement: S7 Fig — Full-length GFP tagged BICD2 WT and the F739I/R743C variant were transiently transfected into HeLa cells that were fixed and immunostained with antibodies against GFP, the G2 phase marker Cyclin B1 and the dynein heavy chain and representative images of WT and F739I/R743C expressing G2 HeLa cells are shown in panels (A) and (B), respectively. While BICD2 WT clearly localized to the nuclear envelope (NE) (A), the F739I/R743C variant showed more diffuse staining at the nuclear envelope in Cyclin B1 positive cells (B). Consistently, the dynein heavy chain staining shows clear NE localization in the WT, but more diffused in the F739I/R743C. Scale bar = 10 μm. Methods: Effectene reagent (QIAGEN) was used for transfection of GFP-BICD2 plasmids [14] in HeLa cells as described in [14]. The medium was replaced after 6 h. Cells were incubated for 1 hr. in Nocodazole (10 μM) prior to fixation, which was performed 24 h post-transfection. For immunostaining, cells were washed in PBS, fixed in -20°C methanol for 10 min, washed in PBS and incubated for 1h with donkey serum in PBS. Immunostaining was performed for 2 h at 37°C with 1:200 dilutions of the following antibodies in blocking solution: Chicken polyclonal antibody against GFP (Millipore AB 16–901), rabbit dynein heavy chain polyclonal #46 antibody [54] and mouse Cyclin B1 antibody (Santa Cruz SC-245). Cover slips were washed with PBS and incubated with 1:200 dilutions in blocking solution of donkey fluorophore-conjugated secondary antibodies (Alexa Fluor 488, Cy3, Alexa Fluor 647, Jackson Immuno Research) for 1 h at room temperature. Cover slips were washed and mounted using AquaPoly/Mount (Polysciences Inc). (TIF) [file pgen.1010642.s007.tif]
